# Effectiveness of Practices To Increase Timeliness of Providing Targeted Therapy for Inpatients with Bloodstream Infections: a Laboratory Medicine Best Practices Systematic Review and Meta-analysis

**DOI:** 10.1128/CMR.00053-14

**Published:** 2015-11-23

**Authors:** Stephanie S. Buehler, Bereneice Madison, Susan R. Snyder, James H. Derzon, Nancy E. Cornish, Michael A. Saubolle, Alice S. Weissfeld, Melvin P. Weinstein, Edward B. Liebow, Donna M. Wolk

**Affiliations:** a Battelle Center for Analytics and Public Health, Atlanta, Georgia, USA; b Centers for Disease Control and Prevention, Center for Surveillance, Epidemiology and Laboratory Services (CSELS), Atlanta, Georgia, USA; c Banner Good Samaritan Medical Center, Banner Health, Phoenix, Arizona, USA, and University of Arizona College of Medicine, Phoenix, and University of Arizona College of Medicine, Tucson, Arizona, USA; d Microbiology Specialists Incorporated, Houston, Texas, USA; e Rutgers Robert Wood Johnson Medical School, New Brunswick, New Jersey, USA; f Geisinger Health System, Danville, Pennsylvania, USA

## Abstract

**Background.:**

Bloodstream infection (BSI) is a major cause of morbidity and mortality throughout the world. Rapid identification of bloodstream pathogens is a laboratory practice that supports strategies for rapid transition to direct targeted therapy by providing for timely and effective patient care. In fact, the more rapidly that appropriate antimicrobials are prescribed, the lower the mortality for patients with sepsis. Rapid identification methods may have multiple positive impacts on patient outcomes, including reductions in mortality, morbidity, hospital lengths of stay, and antibiotic use. In addition, the strategy can reduce the cost of care for patients with BSIs.

**Objectives.:**

The purpose of this review is to evaluate the evidence for the effectiveness of three rapid diagnostic practices in decreasing the time to targeted therapy for hospitalized patients with BSIs. The review was performed by applying the Centers for Disease Control and Prevention's (CDC's) Laboratory Medicine Best Practices Initiative (LMBP) systematic review methods for quality improvement (QI) practices and translating the results into evidence-based guidance (R. H. Christenson et al., Clin Chem **57**:816–825, 2011, http://dx.doi.org/10.1373/clinchem.2010.157131).

**Search strategy.:**

A comprehensive literature search was conducted to identify studies with measurable outcomes. A search of three electronic bibliographic databases (PubMed, Embase, and CINAHL), databases containing “gray” literature (unpublished academic, government, or industry evidence not governed by commercial publishing) (CIHI, NIHR, SIGN, and other databases), and the Cochrane database for English-language articles published between 1990 and 2011 was conducted in July 2011.

**Dates of search.:**

The dates of our search were from 1990 to July 2011.

**Selection criteria.:**

Animal studies and non-English publications were excluded. The search contained the following medical subject headings: bacteremia; bloodstream infection; time factors; health care costs; length of stay; morbidity; mortality; antimicrobial therapy; rapid molecular techniques, polymerase chain reaction (PCR); *in situ* hybridization, fluorescence; treatment outcome; drug therapy; patient care team; pharmacy service, hospital; hospital information systems; Gram stain; pharmacy service; and spectrometry, mass, matrix-assisted laser desorption-ionization. Phenotypic as well as the following key words were searched: targeted therapy; rapid identification; rapid; Gram positive; Gram negative; reduce(ed); cost(s); pneumoslide; PBP2; tube coagulase; matrix-assisted laser desorption/ionization time of flight; MALDI TOF; blood culture; EMR; electronic reporting; call to provider; collaboration; pharmacy; laboratory; bacteria; yeast; ICU; and others. In addition to the electronic search being performed, a request for unpublished quality improvement data was made to the clinical laboratory community.

**Main results.:**

Rapid molecular testing with direct communication significantly improves timeliness compared to standard testing. Rapid phenotypic techniques with direct communication likely improve the timeliness of targeted therapy. Studies show a significant and homogeneous reduction in mortality associated with rapid molecular testing combined with direct communication.

**Authors' conclusions.:**

No recommendation is made for or against the use of the three assessed practices of this review due to insufficient evidence. The overall strength of evidence is suggestive; the data suggest that each of these three practices has the potential to improve the time required to initiate targeted therapy and possibly improve other patient outcomes, such as mortality. The meta-analysis results suggest that the implementation of any of the three practices may be more effective at increasing timeliness to targeted therapy than routine microbiology techniques for identification of the microorganisms causing BSIs. Based on the included studies, results for all three practices appear applicable across multiple microorganisms, including methicillin-resistant Staphylococcus aureus (MRSA), methicillin-sensitive S. aureus (MSSA), Candida species, and Enterococcus species.

## INTRODUCTION

Bloodstream infection (BSI) is a major cause of morbidity and mortality throughout the world (2–5). In 2002, over 30,000 deaths in U.S. hospitals were due to BSIs, and the incidence continues to increase (6). During the period from 2000 to 2010, mortality from septicemia grew by 17% (7), and recent reports still show mortality to range from 34 to 52% (8). In 2009, septicemia, a severe BSI caused by bacteria in the blood, affected nearly 1 out of every 23 hospitalized patients (4.2%) and was the sixth most common reason for hospitalization in the United States (9).

Microorganisms enter the bloodstream through various portals, including dissemination from a previous or concomitant infection and access via surgical sites, intravenous catheters, and other vascular access devices (10). Bloodstream infections can be caused by a wide variety of microorganisms, commonly Escherichia coli, Klebsiella spp., Staphylococcus aureus, Enterococcus spp., other **bacteria, and yeast. These infections can lead to increased mortality, long-term disability, excess length of stay (LOS) in hospitals, large additional costs for health systems, and high costs as well as loss of quality of life for patients and their families. For example, septicemia was the single most expensive condition at U.S. hospitals in 2009, with an aggregate cost of $15.4 billion (9) or 4.3% of all hospital costs. The effect of nosocomial bloodstream infections indicates that health care-associated BSIs result in an additional LOS of over 10 days (11).

Rapid identification of bloodstream pathogens is a laboratory practice that supports rapid transitions to direct targeted therapy, leveraging results to support timely and effective patient care. In fact, the more rapidly appropriate antimicrobials are prescribed, the lower the mortality for patients with sepsis (12–14). Rapid identification methods may have multiple positive impacts on patient outcomes, including reductions in mortality, morbidity, hospital LOS, antibiotic use, and health care expenses (15–17).

## QUALITY GAP: DELAYS IN IDENTIFICATION OF BSIs

Traditional identification and antimicrobial susceptibility test (AST) results for the microorganisms causing BSIs can take 48 h or longer to obtain (18). Immediately after blood is collected for culture, empirical and often broad-spectrum antimicrobial therapy is initiated in patients suspected of having a BSI (19) and continued until the etiological agent is identified and AST results are available to target (tailor) therapy (20). Delay in microbial identification usually results in a lack of timely change from broad-spectrum antimicrobials to targeted therapy. Studies show that up to 40% of patients with BSIs, 50% of those with a health care-associated BSIs, and up to 70% of those with fungemia receive incorrect therapy during the empirical treatment period before the microbiology culture results are available (21–25). Incorrect continuous treatment with broad-spectrum antimicrobials can lead to drug toxicity, antimicrobial drug resistance, increased LOSs, and additional costs for patients and the health care system (26–31). Therefore, efficient communication of the results of Gram staining, microorganism identification, and ASTs that result in timely switches from empirical therapy to targeted therapy are essential for providing safe, effective, and efficient care of patients with BSIs (32, 33).

To reduce this important quality gap and potentially improve patient care, it is essential to identify effective practices that rapidly identify the microorganisms causing BSIs so that timely targeted therapy can be initiated. A systematic review of the effectiveness of rapid diagnostic practices in improving the timeliness of targeted therapy and outcomes in patients with BSIs has not been completed to date. The purpose of this review is to evaluate the evidence for the effectiveness of three rapid diagnostic practices in decreasing the time to targeted therapy for hospitalized patients with BSIs by applying the systematic review methods for quality improvement practices of the Centers for Disease Control and Prevention's (CDC's) Laboratory Medicine Best Practices Initiative (LMBP) and translating the results into evidence-based guidance (1).

## PRACTICE DEFINITIONS

Five practices were initially considered for this review. Based on the available published manuscripts and gray literature, three rapid diagnostic practices were fully evaluated in comparison to conventional 24-h (or greater) microbiology culture with routine reporting methods for results issued after a positive blood culture is identified (i.e., after it “flags positive”). These practices are as follows: (i) rapid molecular techniques with additional direct communication of test results to clinicians or pharmacists to immediately confirm targeted therapy or switch patients from broad-spectrum or empirical therapy to targeted therapy based on the BSI agent, (ii) rapid molecular techniques without additional direct communication of test results (i.e., with only routine communication via an electronic medical record), and (iii) rapid phenotypic techniques with additional direct communication of test results to clinicians or pharmacists.

A “rapid” method was defined as a technique performed on positive blood culture bottles producing results in ≤8 h. This definition was based on the workgroup's decision to assess only rapid FDA-cleared assays and documented references to “rapid” diagnostic tests, defined by the CDC (34) and the World Health Organization (WHO) (35) as those that can be completed in ≤2 h, start to finish. This 8-h limit was selected by combining definitions with the workgroup's understanding of clinical laboratory workflow. In clinical laboratories, the workflow for even the most rapid of test methods, if most likely forced into some form of “batch” testing, is performed one or more times per 8-h work shift due to financial and/or operational considerations. Thus, for the purposes of this review, our practical working definition of a rapid diagnostic tool was one that delivers results in ≤8 h. Point-of-care tests, generally defined as those delivering results within ≤20 to 30 min of specimen collection, were not included, and notably, none existed for bloodstream infections at the time of the review or at the time of this publication. Rapid methods for mass spectrometry were originally included in the literature review but were excluded when none were identified by 2011.

Rapid molecular techniques that met the definition were identified and include the use of PCR and peptide nucleic acid fluorescent *in situ* hybridization (PNA-FISH). Rapid phenotypic techniques, adapted from their intended use to support rapid identification of bloodstream pathogens, were also identified and include tests such as those for tube coagulase (36, 37) and penicillin binding protein 2a (PBP2a) (36, 37), the bioMérieux Vitek 2 system (36, 37), the API 20E bacterial identification system (36, 37), thermonuclease testing (36, 37), and other applicable techniques (36, 37). Both molecular and phenotypic methods reviewed provide microbial characterization above that provided by routine generic Gram stain morphology, which is the historical reference standard (36, 37). The basis for the phenotypic and molecular methods and reports of their performance are published elsewhere (38–42).

For practices 1 and 3, the communication of test results involves directly reporting the results from the rapid identification technique to a responsible clinician or pharmacist who could take action to change therapy based on the laboratory result. These reporting efforts were defined as those that went above and beyond routine result reporting techniques, which included critical value reporting of the Gram stain results as well as reporting final microbial identification through the computer-based laboratory information system. Reporting rapid results generally involved phoning them in as soon as the identifications or AST results for the samples were available (the possibility of reports to nursing or other health care staff as intermediate messengers was not excluded).

Two additional practices were considered in this review, but insufficient evidence, in the form of published articles or unpublished data, was found to evaluate their impact. They were rapid Gram staining (43) and rapid phenotypic techniques with routine communication of test results (4). For the purposes of this review, rapid Gram staining was defined as a situation in which Gram staining is performed and caregivers are informed of the results <1 h after the blood culture signals positive growth.

In summary, for the literature review, point-of-care testing with results in <15 min was applicable; Gram stain results were considered applicable between 15 and 60 min after a flag, and rapid results were defined as 1 to 8 h after a blood culture flag; anything that took longer than 8 h after a flag was not considered rapid for the purposes of this review.

## METHODS

The evidence review for this work followed the CDC's LMBP “A6 cycle” systematic review methods for evaluating quality improvement practices, reported in detail elsewhere (1). This approach is derived from previously validated methods and is designed to evaluate the results of studies of practice effectiveness to support evidence-based best-practice recommendations. As in all A6 cycle reviews, a systematic review question is selected. Appendix 1 provides a list of the data elements of interest for the question posed for this review. Appendix 2 is a glossary of terms used during this process.

Using this method, a review coordinator and staff trained to apply the LMBP methods conducts the systematic review with guidance from an expert panel. The expert panel includes seven to nine members selected for their diverse perspectives and expertise on the review topic. At least one member is an expert in evidence review methodologies. Appendix 3 lists the seven expert panel members participating in this review.

The expert panel reviews the results of the evidence review and drafts the evidence-based best-practice recommendations, which are approved by the LMBP Workgroup. The LMBP Workgroup is an independent, multidisciplinary group composed of 15 members, 13 invited and 2 *ex officio* representatives, from federal agencies (the Center for Medicare and Medicaid Services and the Food and Drug Administration) and with expertise in laboratory medicine, clinical practice, health services research, and health policy. A list of the members of the LMBP Workgroup is provided in Appendix 4.

## ASK: REVIEW QUESTION AND ANALYTIC FRAMEWORK

**FIG 1:**
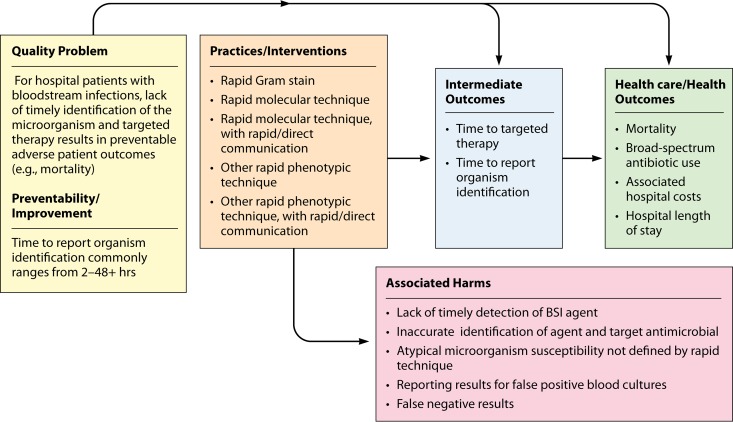
LMBP QI analytic framework: bloodstream infection evidence review question. For hospital inpatients who are admitted for, or are found to have, bloodstream infections (e.g., positive blood cultures), what practices are effective at increasing the timeliness of providing targeted therapy to improve clinical outcomes?

The question to be answered by this evidence review is, “for hospital inpatients who are admitted for, or found to have, bloodstream infections (e.g., positive blood cultures), what practices are effective at increasing the timeliness of providing targeted therapy?” This review question is addressed in the context of the BSI analytic framework depicted in Fig. 1. The relevant population, intervention, comparison, and outcome (PICO) (1) elements are as follows.

- Population: all hospital inpatients who have a BSI
- Interventions:
  - Rapid molecular technique, with additional direct communication
  - Rapid molecular technique, with no additional direct communication
  - Rapid phenotypic technique, with additional direct communication
  - Rapid phenotypic technique, with no additional direct communication
  - Rapid Gram stain
- Comparison:
  - Conventional microbiology testing with phenotypic biochemical or antigenic methods
- Outcomes:
  - Time to targeted therapy is the primary outcome of interest
  - Secondary outcomes (as described below)

For the practices included in this review, “no additional direct communication” was defined as “routine communication” practices, such as those used for reporting of critical values. In contrast, several of the included studies introduced a supplemental rapid communication practice in concert with the adoption of the rapid testing method. These supplemental communication techniques are described below in the descriptive findings. For the latter studies, the effect of rapid testing could not be disentangled from the effect of the simultaneously introduced rapid communication. These results are therefore presented separately from the rapid-testing-only findings.

Although the time to initiate targeted therapy (directed specifically toward the microorganism causing the BSI) was considered to be the most direct (proximal) indicator of the effectiveness of these practices (and thus the primary outcome of interest), other outcomes were also included in this evidence review. Studies that did not include a time to targeted therapy outcome but which included the time to report an identification and/or AST results were included and considered to have a directly relevant outcome.

The expert panel also recommended using the findings for mortality for studies that did not report patient outcomes for either the time to targeted therapy or the time to report testing results. Although the most clinically relevant, mortality is a distal measure of the effectiveness of the rapid identification of BSI agents, as it is influenced by many factors beyond implementation of the rapid test practice. Study quality ratings were thus downgraded for studies contributing only a mortality outcome without statistically accounting for other characteristics of the patient or his/her treatment.

In addition to these findings, results for several other measures have been reported. For example, LOS is a commonly used outcome measure but was considered by the expert panel to be affected by too many medical and nonmedical factors to be considered a proximal measure of rapid-testing effectiveness. Results for these outcomes (i.e., LOS, broad-spectrum antimicrobial use, and cost) are reported in the individual study evidence summary tables provided in Appendix 5.

Practice definitions encompass a range of methods for microorganism identification by phenotypic or molecular practices. Evidence of effectiveness was considered across all microorganisms for a particular practice for which reagents were developed and applicable. Due to the limited availability of published and unpublished data, this approach was maintained for all included studies.

## ACQUIRE: LITERATURE SEARCH AND REQUEST FOR UNPUBLISHED STUDIES

A comprehensive literature search was conducted to identify studies with measurable outcomes. With input from the expert panel and the assistance of a research librarian, a literature search strategy and terms were developed. In July 2011, we conducted a search of three electronic bibliographic databases (PubMed, Embase, and CINAHL) and gray-literature sources and databases, including the International Network of Agencies for Health Technology Assessment (INAHTA), American Medical Association Clinical Practice Improvement and Patient Safety, American Hospital Association, American Medical Association Site Search, American Nurses Association, Canadian Thesis catalog, Canadian Institute for Health Information (CIHI), DART-Europe E-theses Portal, European Health Care and Hospital Federation—Activities, Google Blog search, HealthIT.hhs.gov NIHR Health Technology Assessment Programme, NLM Gateway, Open Gray, Proquest Dissertation Express, Scottish Intercollegiate Guidelines Network (SIGN), Surviving Sepsis Campaign, United Kingdom Clinical Research Network Study Portfolio, Virginia Henderson International Nursing Library (VHINL), and Cochrane database for English-language articles published between 1990 and 2011. Animal studies and non-English publications were excluded.

The search contained the following medical subject headings: bacteremia; bloodstream infection; time factors; health care costs; length of stay (LOS); morbidity; mortality; antimicrobial therapy; Meta-analysis; Review; Evaluation Studies, Clinical Nursing Research Costs; Cost analysis; Cost-Benefit analysis; Nursing; Diagnostic Techniques and Procedures; Diagnosis; Validation studies, Evaluation Studies, Comparative studies; technical report; PNA-FISH; peptide nucleic acids; economics; epidemiology; Outcome assessment; Bacterial Typing techniques; rapid molecular techniques, polymerase chain reaction (PCR); *in situ* hybridization, fluorescence; treatment outcome; drug therapy; patient care team; pharmacy service, hospital; hospital information systems; Gram stain; pharmacy service; mass spectrometry; Matrix-Assisted Laser Desorption-Ionization time of flight; phenotypic; and phenotype. We also included the key words cooperative behavior; agar; targeted therapy; rapid identification; rapid; Gram positive; Gram negative; reduce(ed); cost(s); pneumoslide; PBP2; tube coagulase; thermonuclease; Matrix-assisted laser desorption/ionization time of flight; MALDI TOF; blood culture; EMR; electronic reporting; call to provider; collaboration; pharmacy; laboratory; bacteria; yeast; ICU; and microbiology.

In addition to performing the electronic search, we made a request for unpublished quality improvement data through contacts of the expert panelists as well as e-mails to ASM's ClinMicroNet listserv and the Association of Molecular Pathology's champ listserv; in addition, a general request was posted to the LMBP website, now hosted at http://wwwn.cdc.gov/futurelabmedicine/default.aspx. The website provides instructions for submitting quality improvement data for LMBP reviews.

## APPRAISE: SCREEN AND EVALUATE INDIVIDUAL STUDIES

At least two independent reviewers conducted an initial screening of titles and abstracts of published articles and reviewed full articles and unpublished data submissions to assess eligibility for inclusion for each study. The initial screening of titles and abstracts was used to exclude obviously ineligible studies from a full review. A study was included if it was considered likely to provide valid and useful information and met the PICO criteria previously discussed. Specifically, these inclusion criteria required that a study evaluate a specific intervention/practice included in this review with at least one finding for a relevant outcome measure (i.e., a change in the time to targeted therapy, a change in reporting time, and others noted previously) in a format which was useful for statistical analysis. Studies that did not meet the inclusion criteria (i.e., were not considered studies or did not include a practice of interest or an outcome measure of interest) were excluded from further review.

Published articles and unpublished quality improvement studies retrieved for the review were screened and evaluated by at least two independent reviewers to reduce subjectivity and potential bias. Differences in study quality ratings for each study were resolved through consensus. In addition, five microbiologists, including members of the expert panel and associated members of the American Society for Microbiology reviewed and evaluated the contributing studies. For eligible articles, information on study characteristics, interventions, outcome measures, and findings of the study were extracted using a standardized form. Each study was assigned one of three quality ratings (good, fair, or poor) based on the review of study characteristics and dimensions and assigned one of three effect size ratings (substantial, moderate, or minimal/none) based on the differences in relevant outcome measures after the implementation of the practice. Details on the rating process of individual studies can be found elsewhere (1). Studies that did not meet the LMBP study quality criteria (i.e., those with a fair or good quality rating) were excluded. Data from published studies and unpublished quality improvement projects that passed a full review were transformed to a standardized, common metric according to LMBP methods (1).

## ANALYZE: DATA SYNTHESIS AND STRENGTH OF THE BODY OF EVIDENCE (META-ANALYSIS APPROACH)

The study quality and effect size rating results from eligible individual studies for each practice were aggregated into a practice body of evidence. When possible, an overall summary effect size was calculated to translate systematic review results into one of three evidence-based recommendations (recommended, no recommendation for or against, or recommended against). Both qualitative and quantitative analyses were used to assess the effect size, consistency, and patterns of results across studies (44) and to rate the overall strength of the body of evidence for practice effectiveness (high, moderate, suggestive, and insufficient). Criteria for these ratings are described in greater detail elsewhere (1).

While recommendations are based on the entire body of evidence, effect sizes were calculated for all findings providing sufficient data to estimate the expected impact of a practice. Findings based on continuous data (e.g., the time to report) were standardized using the standardized difference in means (45, 46). Dichotomous findings (e.g., yes/no data, such as mortality) were summarized using the odds ratio (OR) (47).

The quantitative analysis uses the inverse-variance weighted effect sizes from conceptually similar individual studies to produce an overall average weighted effect size (grand mean) and 95% confidence interval (CI). The grand mean is estimated using a random-effects model, and it and the contributing estimates are presented in forest plots which graphically display each study's effect size so that they can be easily reviewed and compared. The *I*^2^ statistic estimates the percentage of variability associated with between-study differences (45, 46).

In addition to there being an interest in evaluating the practices noted here, there was an interest in evaluating whether the effectiveness of rapid testing on the timeliness of the initiation of targeted therapy is related to the effectiveness of rapid testing on mortality. To do this, all effectiveness estimates of timeliness (*d*-scores) were regressed on all effectiveness estimates of mortality (log odds ratios). Although correlations do not inherently prove that causality exists, they are sufficient for estimating whether the two outcomes are likely related and determining whether there is support for the use of mortality as a proxy for timeliness.

## RESULTS OF STUDIES

**FIG 2:**
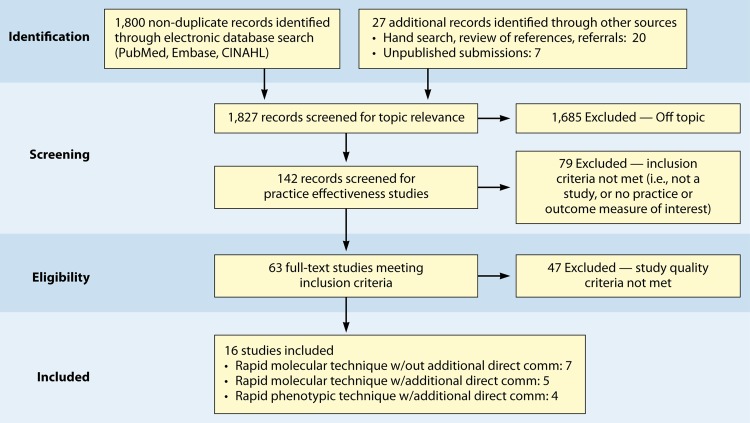
Systematic-review flow diagram. w/, with; comm, communication.

We identified a total of 1,820 nonduplicate bibliographic records and received 7 unpublished submissions covering the period of time between 1990 and July 2011. The reduction in the number of studies through the screening process is detailed in Fig. 2. The most common reason for exclusion of published or unpublished data was the fact that the publication was “off topic”; the second-most-common reason was a lack of applicable practice or outcome data. A total of 16 eligible studies (12 published and 4 unpublished) were considered in the review of practice effectiveness (5 published and 2 unpublished for rapid molecular techniques, 3 published and 2 unpublished for rapid molecular techniques with additional direct communication, and 4 published for phenotypic techniques). Appendix 5 provides abstracted, standardized information and study quality ratings in evidence summary tables for the eligible studies.

There was insufficient evidence to include the practices of phenotypic techniques without additional direct communication and Gram staining in the systematic review evaluation. No studies examining the effectiveness of rapid phenotypic techniques without additional direct communication were located.

Note that the Gram stain technique was used as a routine microbiology test in all of the included studies to confirm the positive blood culture result from the automated continuous-monitoring system; however, only one publication on the use of rapid Gram staining which met the LMBP systematic review study criteria was identified (43). Thus, analytic methods could not be performed. The Barenfanger et al. study (43) is mentioned here to emphasize the potential advantage of rapid Gram staining and reporting of blood cultures as soon as they are flagged positive on the automated continuous blood culture systems. According to this one study, rapid Gram stain reporting has the potential to decrease the time to targeted antimicrobial therapy and decrease morbidity/mortality, the length of a hospital stay, and other associated hospital costs (43). Further studies of this practice are needed to more fully understand and evaluate the effectiveness of this technique. Evidence for the three remaining practices is described below.

### Rapid Molecular Techniques without Additional Direct Communication

Seven eligible studies were found comparing rapid molecular techniques without additional direct communication with conventional microbiology practices with routine communication. These rapid molecular techniques allowed for direct detection and susceptibility testing of bloodstream infections from positive blood culture bottles. The studies included in the final analysis are summarized in Tables 1 and 2. The publication dates of these studies range from 2003 (48) to 2010 (49, 50). Two of the seven studies were originally unpublished (51; K. Stellrecht, M. Grifasi, E. Graffunder, and T. Lodise, unpublished data) (see Table A7 in Appendix 5), and the Frye et al. manuscript was subsequently published and is referenced here (51). Of the seven studies, three were rated “fair” (48, 50; Stellrecht et al., unpublished) (see Table A7 in Appendix 5) and two studies were rated “good” (49, 51). Two additional studies were rated “poor” (52, 53) and were excluded from consideration in this review. All five studies included in the practice body of evidence for this review used PCR molecular techniques to identify BSI agents (48–51; Stellrecht et al., unpublished) (see Table A7 in Appendix 5). Each of the PCR studies used a different technique. All of the studies were performed in the United States using adult inpatients in large (generally with >500 beds) academic teaching or tertiary-care medical centers.

**TABLE 1:**
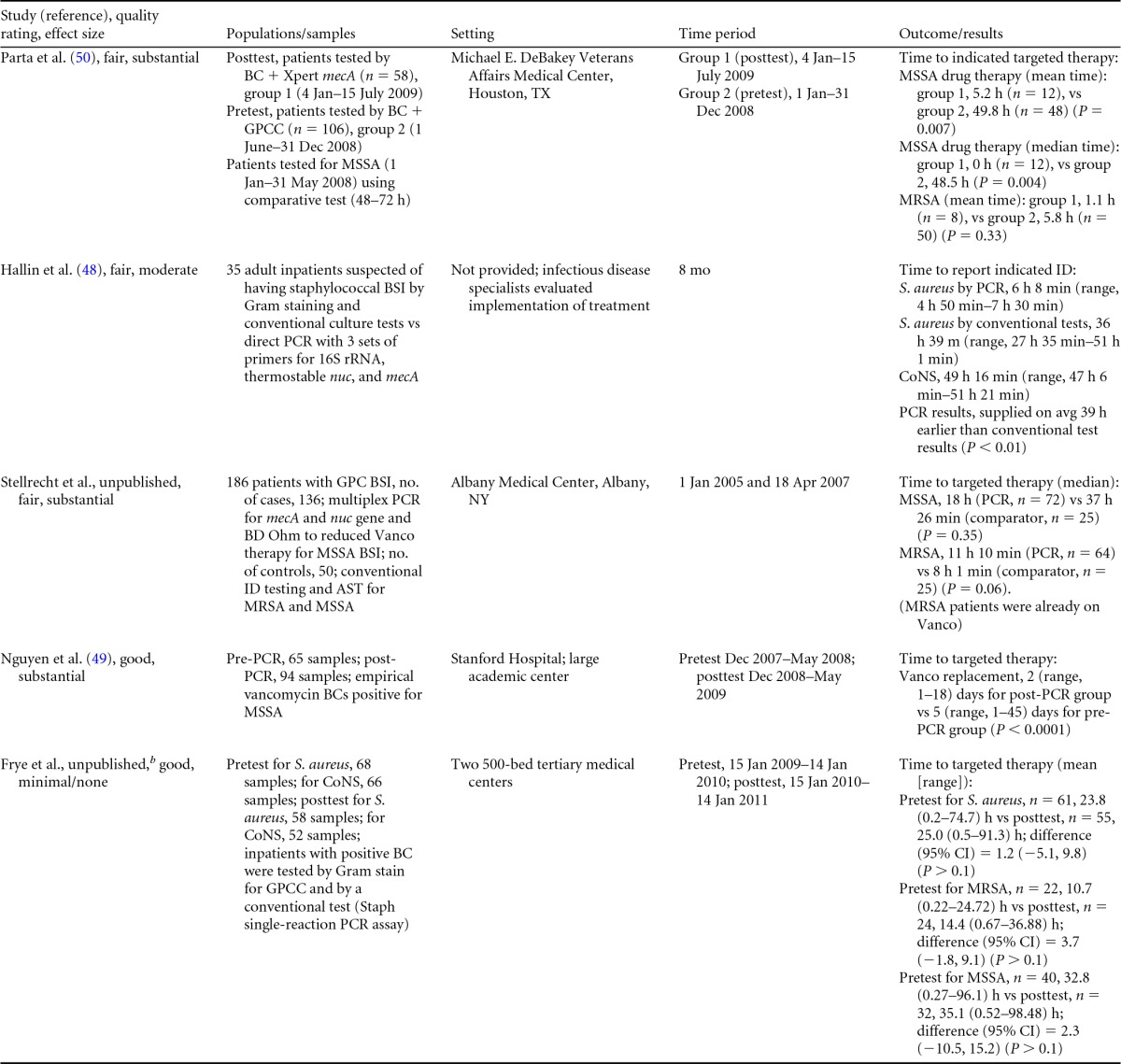
Body-of-evidence summary of rapid molecular techniques without additional direct communication^*a*^

a BC, blood culture; GPC, Gram-positive cocci; GPCC, Gram-positive cocci in clusters; MSSA, methicillin-sensitive Staphylococcus aureus; ID, identification; CoNS, coagulase-negative Staphylococcus; Vanco, vancomycin; AST, antimicrobial susceptibility test.

b Now published as reference 51.

**TABLE 2:** Body-of-evidence LMBP summary ratings for the rapid molecular technique without additional direct communication^*a*^

Study (reference)	Study quality rating	Effect size ratings
Parta et al. (50)	Fair	Substantial
Hallin et al. (48)	Fair	Substantial
Stellrecht et al., unpublished	Fair	Substantial
Nguyen et al. (49)	Good	Substantial
Frye et al., unpublished^*b*^	Good	Minimal/none

a There was one study for which the quality rating was good and the effect size substantial, one study for which the quality rating was good and the effect size minimal or none, and three studies for which the quality rating was fair and the effect size substantial. There was no consistency among them, and the overall strength of the studies was low.

b Now published as reference 51.

#### Body-of-evidence qualitative analysis.

The evidence of practice effectiveness for improving treatment timeliness by rapid molecular techniques without routine communication for hospital inpatients indicates that treatment is inconsistent but often substantially faster than after standard testing (Table 2). One study (Stellrecht et al., unpublished) (see Table A7 in Appendix 5) provided results that could not be standardized. The weighted difference in median hours to the time of targeted therapy for this study was 14.7 h for the rapid molecular technique versus 22.6 h for conventional testing, which was rated as having a substantial effect size. Results for the remaining four studies could be standardized. The standardized differences in means (*d*-score) that were calculated for these four included studies ranged from 0.131 to −0.675 (with negative values favoring the rapid molecular technique over standard testing). The *d*-score for three of the four studies exceeded −0.6, for a substantial effect size rating. Converting a *d*-score of 0.6 into the common-language statistic (54), a randomly selected rapid molecular test result will be reported faster than a randomly selected standard test result approximately 66.4% of the time.

#### Meta-analysis.

**FIG 3:**
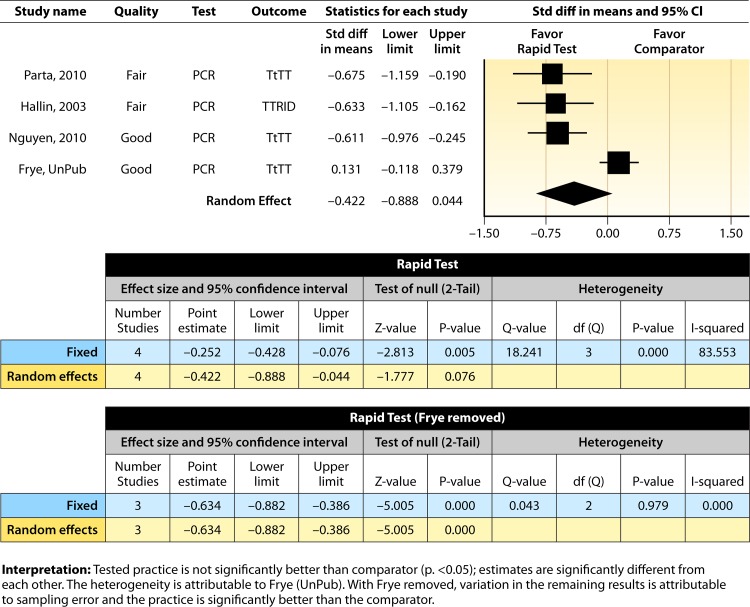
Meta-analysis forest plot showing an analysis of rapid molecular techniques versus routine microbiological methods. The rapid molecular technique was without additional direct communication. Parta, 2010, see reference 50; Hallin, 2003, see reference 48; Nguyen, 2010, see reference 49; Frye, UnPub, Frye et al., unpublished (now published as reference 51); TtTT, time to targeted therapy; TTRID, time to report identification; Std diff, standard difference.

The forest plot in Fig. 3 presents the meta-analysis effect size results for a rapid molecular technique without additional direct communication compared to standard testing for the four studies with standardized results in the body of evidence estimated using a random-effects model. The *d*-score confidence interval suggests that rapid molecular testing without direct communication is not significantly better in increasing timeliness than standard testing (average *d*-score = −0.422; 95% CI, −0.888 to 0.044; *P* > 0.05); however, there is significant heterogeneity in the estimates summarized (*Q* = 18.24, *P* < 0.05), with approximately 84% of the variability in results attributable to between-study differences (*I*^2^ = 83.55) (54). The observed heterogeneity is caused by the work of Frye et al. (51), whose results favored the comparison practice. With the paper by Frye et al. (51) removed, the results of the remaining three studies for a rapid molecular technique are homogeneous (*Q* = 0.043, *P* > 0.05) and the overall improvement in timeliness from implementing rapid molecular testing is statistically significant (mean *d*-score = −0.634; 95% CI = −0.882 to −0.389; *P* < 0.001).

### Rapid Molecular Techniques with Additional Direct Communication

There were five studies on the effectiveness of rapid molecular techniques with additional direct communication practice, which are summarized in Tables 3 and 4. Three of the studies were published (55–57) and two were unpublished (58; D. C. Gamage, D. P. Olson, N. N. Whitfield, L. H. Stickel, D. R. Johnson, and K. R. Matthais, unpublished data) (see Table A12 in Appendix 5). The Heil et al. data were subsequently published, and the report is referenced here (58). Three of the five studies utilized the PNA-FISH method on blood samples from bottles positive for culture as detected by continuous-monitoring culture systems. The PNA-FISH technique in these studies was used to identify Candida (58; Gamage et al., unpublished) (see Table A12 in Appendix 5) and two species of Enterococcus (E. faecalis and E. faecium) (56; Gamage et al., unpublished) (see Table A12 in Appendix 5). One study (55) using the GeneXpert real-time PCR platform identified methicillin-resistant S. aureus (MRSA). All studies were performed in large medical centers in the United States (55–57; Gamage et al., unpublished) (see Table A12 in Appendix 5). One study (55) was rated “good,” and four studies were rated “fair” (56–58; Gamage et al., unpublished) (see Table A12 in Appendix 5). The additional direct-communication interventions for antimicrobial therapy switches were initiated by infectious disease pharmacists (55), laboratory clinical liaisons (57), an antimicrobial management team (56), laboratory staff calling results to infectious disease pharmacists (59; Gamage et al., unpublished) (see Table A12 in Appendix 5), and laboratory personnel paging on-call pharmacists, who then made a recommendation to a medical service based on the institution-specific antibiogram (58).

**TABLE 3:**
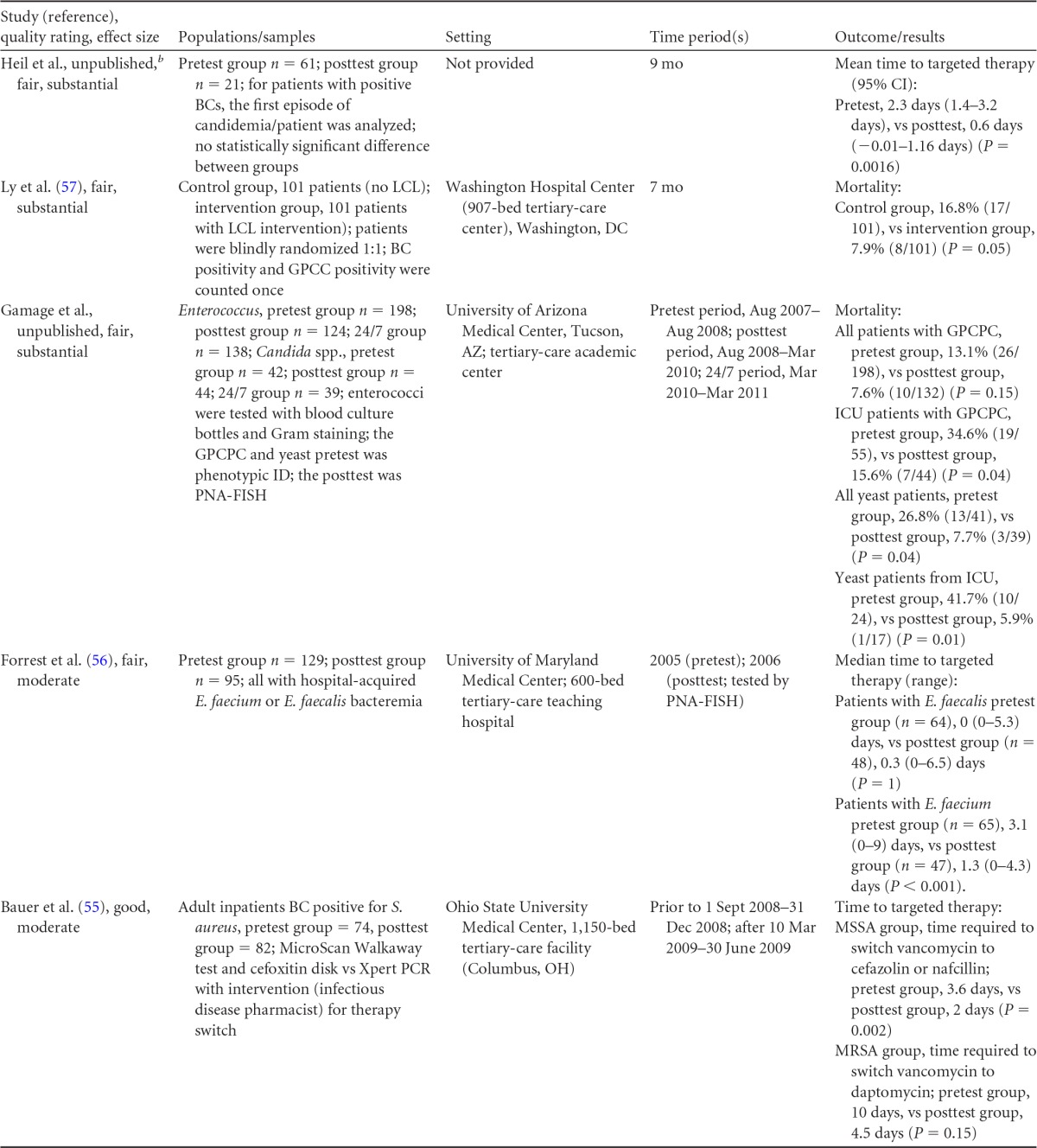
Body-of-evidence summary for rapid molecular techniques with additional direct communication^*a*^

a LCL, laboratory/clinician liaison; GPC, Gram-positive cocci; GPCC, Gram-positive cocci in clusters; GPCPC, Gram-positive cocci in pairs and chains; 24/7, monitored 24 h per day, 7 days per wk; ICU, intensive-care unit.

b Now published as reference 58.

**TABLE 4:** Body-of-evidence LMBP summary ratings for the rapid molecular technique with additional direct communication^*a*^

Study (reference)	Study quality rating	Effect size rating
Heil et al., unpublished^*b*^	Fair	Substantial
Ly et al. (57)	Fair	Substantial
Forrest et al. (56)	Fair	Moderate
Gamage et al., unpublished	Fair	Substantial
Bauer et al. (55)	Good	Moderate

a There was one study for which the quality rating was good and the effect size moderate, three studies for which the quality rating was fair and the effect size substantial, and one study for which the quality rating was fair and the effect size moderate. There was no consistency among them, and the overall strength of the studies was low.

b Now published as reference 58.

#### Body-of-evidence qualitative analysis.

Primarily due to the “fair” quality of eligible studies, the strength of the evidence that timeliness of treatment in hospital settings is improved using rapid molecular techniques with additional direct communication compared to standard testing without direct communication is low (Table 4). Of the five studies identified, three provided results directly assessing the time to targeted therapy. The standardized difference in means (*d*-scores) for these studies ranged from −4.023 to −0.351 (with values less than 0.0 favoring a rapid molecular technique with direct communication over standard testing and standard communication). Two additional studies (57; Gamage et al., unpublished) (see Table A12 in Appendix 5) provided results documenting substantial reductions in mortality when rapid molecular testing with direct communication was implemented (OR = 0.425 [95% CI, 0.174 to 1.036] and OR = 0.576 [95% CI, 0.314 to 1.058], respectively).

#### Meta-analysis.

**FIG 4:**
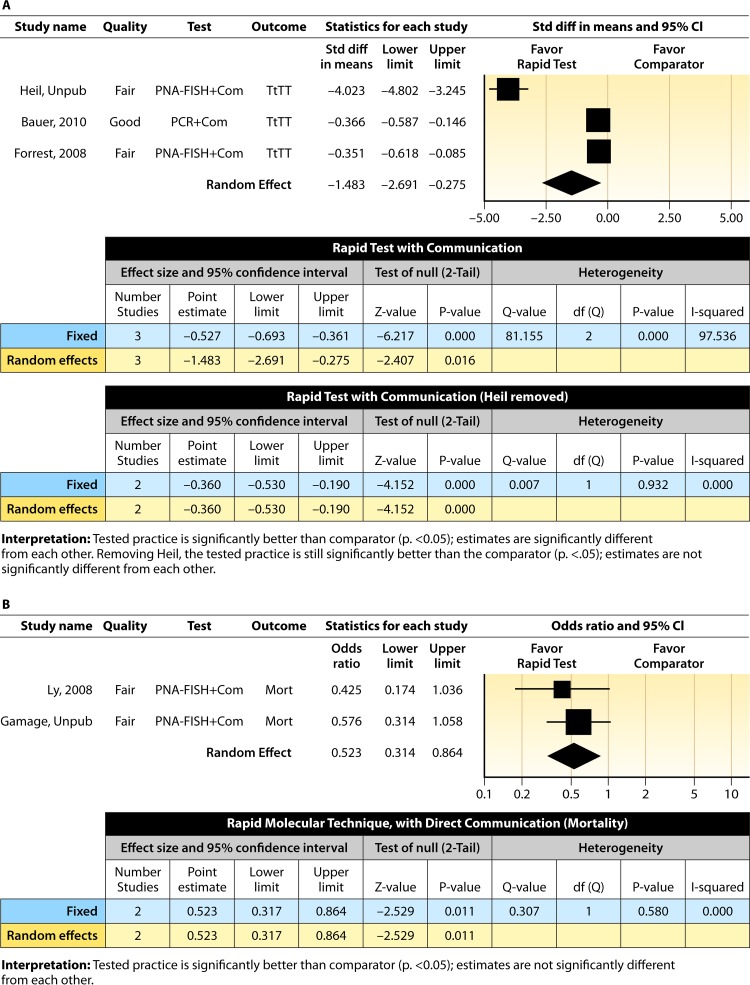
(A) Meta-analysis forest plot of rapid molecular techniques, with additional direct communication, versus routine microbiological methods. Heil, Unpub, now published as reference 58; Bauer, 2010, see reference 55; Forrest, 2008, see reference 56; Com, comparator. (B) Meta-analysis forest plot of a rapid molecular technique, with additional direct communication, versus routine microbiological methods of interpretation. Ly, 2008, see reference 57; Gamage, Unpub, Gamage et al., unpublished; Mort, mortality.

The forest plot in Fig. 4A presents the meta-analysis of effect size results estimated using a random-effects model for a rapid molecular technique with additional direct communication compared to standard testing for the three studies with a standardized difference in means in the body of evidence. Combined, the *d*-scores for the three studies suggest that rapid molecular testing with direct communication significantly improves timeliness compared to standard testing (mean *d*-score = −1.483; 95% CI, −2.691 to −0.275; *P* < 0.05). Heil et al.'s (58) rather remarkable effect size (*d*-score = 4.023) creates considerable heterogeneity (*Q* = 81.16; *P* > 0.000). Removing the results of Heil et al. (58) returns a homogeneous distribution (*Q* = 0.007; *P* > 0.05) and a commensurately smaller, albeit still significant, improvement in the timeliness of targeted therapy (mean *d*-score = −0.360; 95% CI, −0.530 to −0.190; *P* < 0.001). With the work of Heil et al. (58) included, a randomly selected rapid molecular test result with direct communication will be reported faster than a randomly selected standard test result approximately 85.3% of the time; excluding the work of Heil et al., the report will be faster approximately 60.1% of the time.

Two studies (57; Gamage et al., unpublished) (see Table A12 in Appendix 5) did not report a timeliness estimate but did provide mortality data (Fig. 4B). Combined, these studies show a significant and homogeneous reduction in mortality associated with rapid molecular testing combined with direct communication (mean OR = 0.523; 95% CI, 0.317 to 0.864; *P* < 0.05; *Q* = 0.307; *P* > 0.05).

### Rapid Phenotypic Techniques with Additional Direct Communication

Information from the four included studies with evidence of the effectiveness of rapid phenotypic techniques for direct detection and susceptibility testing of bloodstream infections from blood culture bottles with additional communication compared to conventional microbiology practices is summarized in Tables 5 and 6. Studies for this practice include identification and susceptibility of a wide range of microorganisms and include specimens in addition to blood culture. The publication dates for these studies range from 1989 (60) to 2008 (61). The quality of all four studies for this practice was rated “fair” (60–63). Two studies were performed in large university medical centers (61, 63), one study was performed in a multisite teaching hospital laboratory setting (62), and the fourth study was performed in a large hospital (60). Two studies were performed in The Netherlands (61, 62), and two were performed in the United States (60, 63). Additional direct communication interventions were initiated by an infectious disease fellow making recommendations directly to the physician in charge (60), by laboratory staff immediately phoning identification and antimicrobial results to an infectious disease consultation service (61) or directly to physicians who had requested the analysis (63), and by a clinical microbiologist phoning clinically relevant information and treatment advice, if necessary, to the attending clinician (62).

**TABLE 5:**
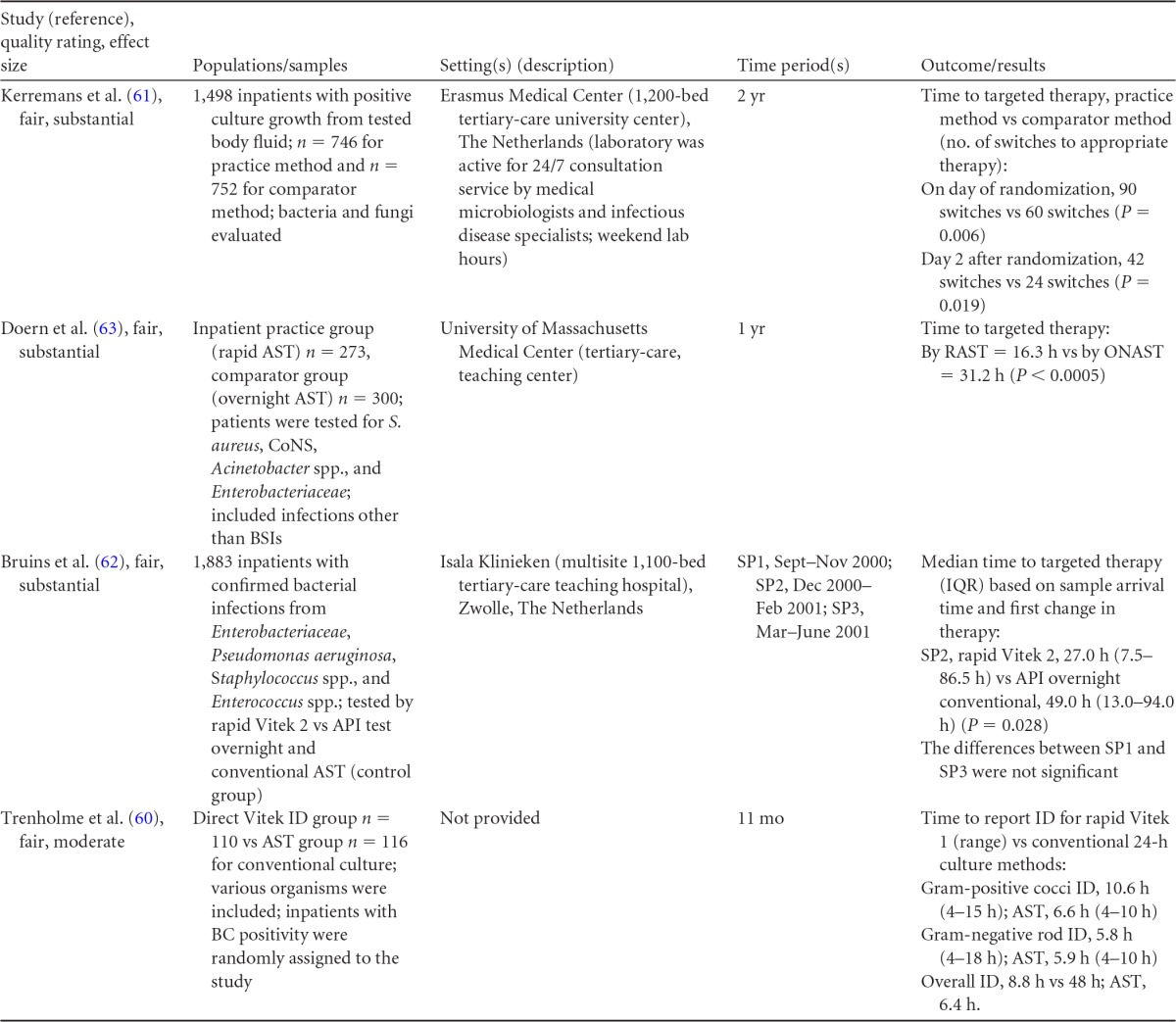
Body-of-evidence summary for the phenotypic technique with additional direct communication^*a*^

a RAST, rapid antimicrobial susceptibility testing; ONAST, overnight antimicrobial susceptibility testing; SP1, -2, and -3, study phases 1, 2, and 3, respectively; IQR, interquartile range.

**TABLE 6:** Body-of-evidence LMBP summary rating for the phenotypic technique with additional direct communication^*a*^

Study (reference)	Study quality rating	Effect size rating
Kerremans et al. (61)	Fair	Substantial
Doern et al. (63)	Fair	Substantial
Bruins et al. (62)	Fair	Substantial
Trenholme et al. (60)	Fair	Moderate

a There were three studies for which the quality rating was fair and the effect size substantial and one study for which the quality rating was fair and the effect size moderate. There was consistency among them, and the overall strength of the studies was low.

#### Body-of-evidence qualitative analysis.

Evidence of effectiveness for improving treatment timeliness using rapid phenotypic techniques with additional direct communication indicates generally substantial improvement over standard testing practices. All four studies were of fair quality, and the three studies assessing time to targeted therapy each reported “substantial” improvement in timeliness (61–63), resulting in an overall low strength of evidence in hospital settings. Trenholme et al. (60) reported a moderate improvement in the time to report an identification. Two of the four studies, those of Kerremans et al. (61) and Doern et al. (63), provided significant positive results in improving timeliness that could be standardized (*d*-scores, −0.270 and −0.146, respectively).

#### Meta-analysis.

**FIG 5:**
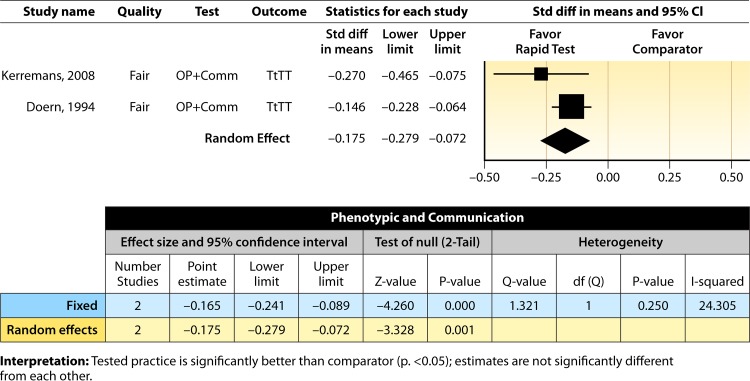
Meta-analysis forest plot of phenotypic techniques, with additional direct communication, versus routine microbiological methods. Kerremans, 2008, see reference 61; Doern, 1994, see reference 63.

The forest plot in Fig. 5 presents the meta-analysis effect size results for rapid phenotypic techniques with additional direct communication compared to standard testing for the two studies with standardized time-to-targeted-therapy outcome measures estimated using a random-effects model. A significant and homogeneous grand mean suggests that rapid phenotypic techniques with direct communication likely improves the timeliness of targeted therapy (average *d*-score = −0.175; 95% CI, −0.279 to −0.072; *P* < 0.001). Converting these to the common-language statistic suggests that a randomly selected rapid phenotypic test result with additional direct communication will result in targeted therapy faster than a randomly selected standard test result approximately 54.9% of the time.

#### Impact on mortality.

**FIG 6:**
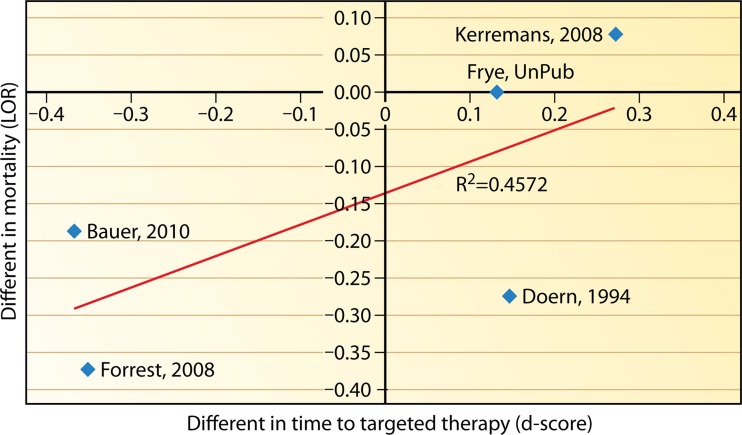
Regression of the effectiveness estimates of mortality (log odds ratios [LOR]) versus effectiveness estimates of timeliness to targeted therapy (*d*-scores).

There is interest in whether the implementation of a rapid technique and communication practice(s) reduces mortality. Five studies included in this review provided estimates for both the time to targeted therapy after implementing a rapid identification technique (with or without additional direct communication) and mortality (51, 56, 61–63). As noted previously, the effectiveness estimates of timeliness to targeted therapy from these studies (*d*-scores) were regressed on the effectiveness estimates of mortality (log odds ratios). Figure 6 shows the resulting regression. Note that one study by Heil et al. (58) was left out of the analysis, as its estimate on timeliness to targeted therapy is an outlier and significantly different from the other reported results. The random-effect results for this regression showed an *r* of 0.6762 (*P* = 0.3366; *r*^2^ = 0.4572). Although a strong correspondence between the timeliness of targeted therapy and mortality can be observed, given the evidence available, the relationship fails to reach significance. Although it seems likely that the two outcomes are associated, that relationship is yet to be proven.

## ADDITIONAL CONSIDERATIONS

This section addresses additional considerations for implementing molecular and phenotypic methods for rapid identification and susceptibility testing with or without direct communication practices for early intervention and administration of targeted therapy to patients with BSIs.

### Applicability

All studies included in this review were conducted on adult inpatients in large (generally >500 beds) academic teaching or tertiary-care medical centers, but observed improvements in times to targeted therapy may be generalizable to other health care settings. Studies in this review included a variety of settings, such as a Department of Veterans Affairs Medical Center and academic tertiary-care centers (50, 55, 56, 59). Study institutions with practices of rapid molecular techniques of PCR and PNA-FISH without additional direct communication practices had routine standards of communication in place similar to those typically found in most hospitals to confirm and switch appropriate antimicrobial therapy for patients with BSIs (48–50). Some sites appeared to have efficient communication without additional direct methods (49) based on described outcome results (Fig. 3). Hospitals implementing the practices of rapid molecular techniques or phenotypic techniques with additional direct communication included multidisciplinary teams with an infectious disease physician(s) and infectious disease pharmacists, research data managers (55), on-call pharmacists (58), and a laboratory/clinician liaison (57), which were essential to the practice.

### Associated Harms

Although not identified in the evidence base, one of several hypothetical scenarios may suggest potential harm from the use of rapid identification techniques. Harms include the lack of timely and accurate detection of a BSI agent, despite rapid testing of positive blood culture bottles. In this scenario, the gains of the rapid testing and reporting would be nullified by the fact that the rapid intervention failed to identify the pathogen; therefore, additional laboratory costs would not be offset by a reduction in nonlaboratory costs. Risks of antimicrobial de-escalation, based on a negative rapid result, may exist during the time interval between the rapid result and the time at which the traditional culture and phenotypic methods produce a final identification and AST results. Furthermore, inaccurate identification of the microorganism might lead to inappropriate and ineffective changes in antimicrobial therapy, which might have significant repercussions on patient health and care if the therapy change is not warranted.

Likewise, a small proportion of blood cultures will yield more than one pathogen per bottle, resulting from infection with more than one pathogen or identification of a pathogen mixed with a typical skin contaminant. In this scenario, if rapid methods were not able to detect or discriminate between multiple pathogens, the outcome would be similar to that of the false-negative result described previously. This harm is partially mitigated by the fact that none of the assays promote rapid testing alone, without the use of a Gram stain, which can help distinguish between some pathogens in mixed infections. Furthermore, risks are limited by the subculture of the blood culture bottles, as subculture can more accurately identify mixed infections than a Gram stain alone. The implications of rapid testing methods described here may change if mixed infections become more common. Routine methods are still the definitive reference standard, and any discrepancies between a rapid method and the definitive culture result should be closely monitored; if antimicrobial therapy was inappropriately altered based on the rapid result, each instance would be reported as a potential patient safety risk.

A second harm may occur if the BSI-causing microbes that were identified by the rapid technique do not behave according to the respective institutional antibiogram. The use of an institution-specific antibiogram to guide therapy after rapid microbial identification to the genus or species level cannot account for exceptions and unforeseen mutations causing atypical susceptibility that the rapid technique cannot define. This circumstance might place patients with BSIs at risk until a final AST report is issued. Of note, this harm is implicit in all rapid methods upon which antimicrobial therapy is based and is also implicit in empirical therapy regimens.

Detection of bloodstream infections by molecular or phenotypic methods also includes the possibility of reporting results for false-positive or contaminated cultures and false-negative results due to insufficient growth in the blood culture itself. Rapid reporting of results by rapid molecular or phenotypic methods may give physicians a false sense of accuracy, causing them to overlook the basic limitations inherent in the blood culture process itself. While false-positive blood culture results are likely to be identified sooner with rapid methods and add value to differential diagnoses, false-negative results must be mitigated by clinical evidence, since some of these organisms do not grow in standard blood cultures without selective culture medium requirements or special growth conditions. False-negative results may also be due to a lack of sensitivity in the test system to detect the low density of some microorganisms.

### Additional Benefits

Studies reviewed suggest beneficial outcomes (decreasing mortality) associated with rapid molecular and rapid phenotypic techniques with or without additional direct communication (17, 52, 55–58; Gamage et al., unpublished) (see Table A12 in Appendix 5) and length of hospital stay (49, 55, 58) in addition to reducing the time to targeted therapy in patients with BSIs. One study showed that PCR surveillance for MRSA in a small community hospital was associated with decreasing the length of stay by 9.3% in the intensive care and critical care units in 2009 (D. Uettwiller-Geiger, unpublished data) (see Table A15 in Appendix 5). Studies reviewed also indicated that the time for the laboratory to report the identification of the microorganism causing the BSI was reduced when rapid molecular or phenotypic techniques were used (17, 48, 51, 56, 61). Several studies also documented that there was reduced use of broad-spectrum antimicrobials when rapid molecular techniques were employed, even in the absence of additional direct communication practices (49, 50, 53, 56, 59). Studies using rapid molecular or phenotypic techniques with additional communication also showed reductions in the use of broad-spectrum antimicrobials (55, 60, 61). Across all of these studies, reductions in the defined daily dose of antimicrobial medications ranged from 20 to 60% with the implementation of the practice.

### Economic Evaluation

Decreasing the time that it takes for BSI microorganism identification and an AST result is commonly considered likely to reduce health care costs for both patients and institutions by reducing the time to appropriate targeted therapy. No economic evaluation studies that comply with guidelines for full economic evaluation (64–66) were found for the rapid testing techniques evaluated in this review. Direct medical costs are impacted by the patient's length of hospital stay, repeated use of laboratory testing or other diagnostic procedures, use of broad-spectrum antimicrobials, use of targeted antimicrobial therapy, and other pharmaceutical costs (28, 57).

Several studies reported a decrease in health care expenses after implementing rapid testing (55, 57, 59, 63; Uettwiller-Geiger, unpublished; Gamage et al., unpublished) (see Table A15 in Appendix 5 and Table A12 in Appendix 5), while others report decreased costs for antimicrobial agents (52, 62) and the total antifungal costs per patient (59). However, the cost reductions reported in these studies do not account for any additional costs associated with implementing rapid testing or costs and benefits associated with nonmedical costs (e.g., patient and caregiver time). Providing rapid testing may require additional laboratory space and additional staffing for both testing and direct communication. Other cost considerations that cannot be appropriately addressed are related to batching of tests, which occurred in most studies to make more efficient use of laboratory resources and reduce overall costs of testing.

### Feasibility of Implementation

Implementation of the practices discussed in this review may be affected by specific hospital environments, laboratory settings, staff competencies, specimen volume, budget considerations, and the ability to provide active notification of test results to clinicians or pharmacists who will provide early antimicrobial interventions and appropriate therapy for patients with BSIs (19, 67). Implementing any new test into a microbiology laboratory or new practice in a hospital setting often encounters resistance due to efforts to control budgets related to reagents, human resources, and other factors (17, 28, 60, 61). Selection of an appropriate laboratory technique that best suits an institution often depends on making a business case, demonstrating potential quality outcomes or cost-effectiveness metrics (68).

A variety of different phenotypic and molecular techniques were utilized in the studies evaluated in this review; however, most of the studies in this review involved molecular tests performed in large university or tertiary-care centers. A number of the techniques have the capacity to identify a range of microorganisms directly from blood culture bottles determined to be positive for bacteria or yeasts (53, 55, 56; Gamage et al., unpublished) (see Table A12 in Appendix 5). The hands-on times, test turnaround times, costs, and types of reagents, as well as technical skills required to perform the test, varied among these different techniques. A single PNA-FISH test process was described and used with different PNA probes and was said to be less expensive and simpler to perform (not requiring special laboratory space) than a number of different PCR procedures with which different equipment, reagents, skill levels, laboratory space, and costs were associated (48–50, 52, 54, 55, 69).

Test sensitivity and specificity are important considerations in determining feasibility for implementing a new diagnostic laboratory procedure. Studies in this review for PNA-FISH and PCR methods provided 95% or greater sensitivity and specificity for detection of the genetic targets (16, 48, 51, 52, 54, 57, 59). A potential advantage of PCR over PNA-FISH is that PNA-FISH requires at least 10^4^ organisms/ml in blood for detection (70), while the limits of detection for PCR are typically, but not always, lower (70–72). Since microbial genetic targets are amplified in PCR, it should be able to detect organisms present at lower microbial densities. A disadvantage of PCR is related to primer specificity and competitive inhibition, which can occur when one target at higher density limits the amplification of the genetic target at lower density. There is no competitive inhibition with PNA-FISH; each microbe can freely bind with a probe on its own and can be visualized independently by fluorescence microscopy. Rapid phenotypic tests have an organism density requirement similar to that of PNA-FISH for appropriate levels of detection and accuracy.

An important aspect of any rapid testing method is to ensure that test results reach the clinician in a timely manner with specific interventions that might improve relevant patient outcomes. For the studies that implemented practices with additional direct communication, various staff that could quickly act on results and affect interventions were needed. For example, studies by Forrest et al. (56) required a hospital-based antimicrobial management team which consisted of a full-time infectious disease pharmacist and an infectious disease physician who devoted 25% of his/her time to antimicrobial stewardship. These types of staff members and teams may not be readily available in all hospitals.

## FUTURE RESEARCH NEEDS

The findings of this systematic review highlight the limited number of good-quality studies evaluating the impact of rapid testing of the microorganisms causing BSIs on reducing the time to targeted therapy for hospitalized patients. For those on-topic quality improvement studies that were conducted and reviewed but not included in the body of evidence, there are a number of reasons they were not eligible for this review. For some, the information provided was insufficient to allow an estimate of the effect of the practices of interest. For example, some studies reported a percentage improvement in timeliness but did not specify the value on which the improvement was based. For others, reported outcomes were only indirectly attributable to the practice (e.g., LOS, broad-spectrum antimicrobial use). While these outcomes may be affected by reduced times to targeted therapy, several other factors also influence these outcomes. The list of proposed study and reporting guidelines in Appendices 1 and 6 can be used to outline the elements recommended for future studies that aim to address the question presented in this systematic review with the rigor required to meet the systematic review inclusion criteria; one should also take into consideration the limitations of the studies identified for this review. Elements of Appendix 1 are discussed in detail below.

Most publications considered for this systematic review did not provide patient or clinical outcome data as a result of the rapid molecular or phenotypic practice(s) performed and were therefore not included in the analysis. Future studies should strive to obtain information on patient-centered outcome results and also to include the most-proximal outcome measures (such as time to targeted therapy and time to report identification results) such that the direct effect of implementing a particular practice may be assessed.

Many of the studies evaluated had small sample sizes, sometimes as few as 30 patients, from which to draw conclusions. Such small sample sizes limit the precision of results and reduce the likelihood that findings are applicable across a larger population (73). It is important that future studies select a sufficiently large sample size to enhance the accuracy and precision of the results observed.

Most of the studies reviewed lacked sufficient information on important study details to meet the inclusion criteria for LMBP systematic review. Had additional information been provided, it is possible that it would have improved study quality ratings. Future studies should include detailed information on topics such as the facility and study setting, sample population description and size, numeric ranges and standard deviations (SD) of results (i.e., not only arithmetic means), detailed descriptions of the comparator and intervention practice, information describing the staff and resources needed to implement the practice, and descriptions of changes in any ancillary practice, procedures, or other health care interventions that may bias study findings. As LMBP systematic reviews focus on the preanalytic and postanalytic phases of testing rather than the analytic phase (1), future studies should identify, address, and discuss the patient outcome quality gap that the laboratory practice is designed to improve.

One hypothesizes that decreasing the time to initiating targeted therapy in patients through the use of rapid molecular or phenotypic techniques in turn leads to lower morbidity and decreased rates of mortality, further supporting the use of (and costs associated with) such practices. However, as discussed previously, limited data were identified in this review to support this hypothesis. We need studies that collect both the immediate and longer-term outcome data necessary to test these relationships and conduct the analyses required to confirm that observed improvements in patient outcomes are attributable to changes in laboratory practices. More research is also needed to encompass the impact of varied hospital settings, such as small or nonacademic institutions, to document the effectiveness of rapid molecular and phenotypic techniques in different settings. A better understanding of how batching and other such routines for testing samples is needed and should also be considered for inclusion in upcoming studies.

Cost-related results should also be reported, based on quality standards for economic evaluations that would make the results meaningful and potentially generalizable and allow for comparisons across studies. A full economic evaluation (64–66) is beyond the scope and the funding of this review, and the concept is being considered for futures reviews. Economic evaluation is a process completely different from that of the LMBP; it is worthwhile, but it does not correspond to the expertise of the LMBP team at this time and is not the purpose of the LMBP question. Guidance on cost information to consider for inclusion in future studies is provided in Appendices 1 and 6.

The body of evidence presented in this review shows that there are not sufficient good-quality studies on any one molecular or phenotypic technique to evaluate the technique's effectiveness alone. Further research on individual techniques and on the effectiveness of these techniques on specific microorganisms is needed. Clearly, more investigation needs to occur as part of the ongoing A6 cycle and the “assess” phase begins anew. While the A6 process does not have accommodations for modifying the original systematic review process and meta-analysis, which generally takes over 1 year to complete, it does accommodate a literature refresh process, which uses the original search terms to identify publications that may fit criteria between the time of the review and the time of manuscript submission. Another full systematic review began again in September 2015. The ongoing process by which the rereview should occur is under development by the CDC and ASM to determine factors that will trigger repeating the entire A6 cycle, including newly designed metrics to describe the uptake of guidelines across the laboratory community. Originally, the CDC felt that the process would occur every 5 years, but for the BSI project, that rereview will occur immediately, completing the A6 process and beginning it anew immediately after this paper went into production.

## LIMITATIONS

This review includes more studies on molecular techniques than it does on traditional phenotypic test procedures commonly used in clinical or diagnostic laboratories. Most molecular procedures can be completed with a turnaround time of 2 to 3 h, but in most studies, turnaround times may be higher, as the tests were batched and performed once, twice (52, 53, 55–57), or three times per day (Gamage et al., unpublished) (see Table A12 in Appendix 5). The primary reason for batching tests appears to be related to a higher cost per test with lower test volume due to additional quality control requirements (49, 54) and limited staffing of medical laboratory scientists during off-shift laboratory work hours (50). Batching tests decreases the benefit of rapid test turnaround times, since delays in performance of the test also delays the reporting of test results to clinicians and hence the opportunity to change from empirical to targeted therapy.

Although some rapid techniques can provide test results in approximately 1 h, timely evaluation of specimens is lacking when technical staff are not available to perform the rapid identification test (43). When rapid test results were available, additional direct communication for antimicrobial intervention from broad-spectrum to targeted therapy was sometimes not available (53); therefore, the true impact of rapid testing may be underestimated.

Several studies were based on small samples (48, 50, 51, 55, 58) and were performed in a single center (55, 56, 59). Molecular practices, such as PCR and PNA-FISH, were performed in large academic tertiary-care medical centers with technologists experienced in molecular techniques (50, 52, 56, 59), which may not be representative of other hospital settings.

Most studies in this review focused on distinguishing methicillin-resistant S. aureus from non-methicillin-resistant Staphylococcus species other than S. aureus (49, 53, 57, 59). A few studies evaluated blood cultures for Enterococcus faecalis and E. faecium (56, 62; Gamage et al., unpublished) (see Table A12 in Appendix 5). The limited number of microbes evaluated is notable, in that not all microbes may be associated with treatment algorithms that would warrant the costs or efforts for their rapid identification.

Most of the studies reviewed used a before/after or pre/postintervention study design. Because of the uncontrolled nature of this quasi-experimental study design, there may have been unmeasured factors that changed between study periods that account for or influence the study results. In many studies, patients were analyzed retrospectively, creating the potential for an information bias in pretest/posttest groups (28, 51, 53; Stellrecht et al., unpublished) (see Table A7 and Appendix 5). Despite this potential design effect, it is recognized that for practical reasons, randomized control clinical trials are beyond the scope of typical laboratory quality improvement studies.

Some of the outcome measures attributed to this topic are proximal measures of effectiveness of rapid identification practices, providing more-direct information on the effectiveness of a given practice (e.g., time to targeted therapy, time to report identification results). Other outcome measures are considered more distal, having multiple steps between the implementation of the practice and the outcome measure itself (e.g., length of stay and mortality).

While the time to report the identification results is considered the most direct measure of the effect of rapid testing, because of the many studies using batch testing or other factors influencing the reporting of test results, the possible improvement in time to targeted therapy reported in this review may be understated. In addition, since it is not uncommon for health care facilities to report blood culture results to nursing staff as an intermediate step before the physician sees the results, our work may include a reporting bias, since none of the publications analyzed reported nursing as their primary point of contact.

Most phenotypic and some molecular techniques in this review used multiplex systems designed to identify and provide susceptibility testing for a wide range of bacteria, including Staphylococcus species other than S. aureus and/or Gram-negative bacilli. Some Enterobacteriaceae, nonfermenting Gram-negative bacilli, and Staphylococcus species took longer to identify than other organisms. A longer time was also required for multiplex molecular techniques, and these factors had some effect on the test turnaround time for identification. Tests were generally batched, and additionally, samples other than blood were included in statistical calculations, as most studies also collected urine, pus, or spinal fluid and often did not separate the findings based on collected specimens.

The LMBP systematic review methods are consistent with practice standards for systematic reviews (1), but all such methods include subjective assessments at multiple points that may produce bias. Rating study quality depends on consensus assessments that may be affected by such things as rater experience and the criteria used. As is the case with most systematic reviews, publication bias must be considered despite the inclusion of unpublished studies, which may help to mitigate that bias. One of the strengths of the LMBP process is the inclusion of nonpublished data to help reduce publication bias. The restriction to English-language studies to satisfy the requirement of multiple reviewers for each study may also introduce bias.

It is commonly known that few if any molecular assays worldwide will totally escape primer redesign or the need to add additional primers, as pathogen genetics tend to change in response to pressures such as antimicrobial use and laboratory testing strategies. The results listed here summarize impacts from pathogen genetics reported up to July 2011.

We acknowledge that other sentinel publications exist, and it is not the intent of the A6 process to limit their value to the microbiology community. However, due to the structure of the question being addressed and of the process structure, several key publications were omitted from analysis but are listed here for the reader (74–78).

While the LMBP process does not make quality judgments based on statistical power or statistical significance, the process does make quality judgments on whether the data used in the study are likely to be representative of the “true” impact of the practice. Many attributes, for example, quality of measurement, the measures used, the span of measurement, and the inclusion/exclusion criteria used in obtaining patients, can influence the representativeness of the results. If a study uses a census and a sufficiently long sampling period but still has few subjects, it is likely to get a good quality score for representativeness of the findings, regardless of the statistical properties of the finding. The effect size is graded separately from the quality of the study in order to produce that effect. There is also a very good paper by Ioannidis (79) worth citing to further describe this concept. The process is not intended to denigrate sentinel papers, nor will it do anything to stop sentinel papers from becoming sentinel papers or being referenced in reviews of the literature, as they should be. This LMBP review simply requires that certain criteria be met to be included in analysis. Definitions of the LMBP criteria are clearly defined and published so that if authors want to be included in the meta-analysis, they have clear guidelines to follow in future publications. In addition, Appendix 6 provides a toolkit or roadmap to assist authors in collecting all the information needed in order to provide data that can be included in a future meta-analysis.

Finally, this work represents not only the results of a meta-analysis, it represents the very first rigorous and programmatic systematic review collaboration among practicing clinical microbiologists, physicians, allied health care personnel, epidemiologists, and biostatisticians aimed to address key questions and controversies in the practice of clinical microbiology. As with any “first,” there are limitations, some of which remain to be uncovered. It is the intent of the A6 process to continually uncover and disclose limitations with full transparency. Anyone can obtain raw data used for this evaluation from the American Society for Microbiology, and updates will be ongoing, as described in “Data Since the Time of Initial Review” below.

## DATA SINCE THE TIME OF INITIAL REVIEW

While we acknowledge that there have been significant technological leaps since the inception of our work, based on the structure of the LMBP process, the new publications cannot contribute to the current document. We reference key publications that support or refute our conclusions and document the fact that these publications will be included in the next systematic review (74–78).

It was not our intent to review assay accuracy data; therefore, many publications, including many citing SeptiFast methods and matrix-assisted laser desorption ionization–time of flight (MALDI-TOF), were excluded because no outcome data relevant to the questions were reported in those papers relevant to the PICO question (1). The LMBP forces standardization of outcome and data points, such that the LMBP meta-analysis does indeed assess defined standard practice and data elements to which all the publications must comply to be included. Few manuscripts meet criteria to make it through the LMBP process; therefore, the process limits the inclusion of many articles. It is the intent of the CDC and ASM to promote transparent publications and data that are more easily comparable with systematic review statistics.

## CONCLUSIONS AND RECOMMENDATION

On the basis of low overall strength of evidence of effectiveness, no recommendation is made for or against the use of the three assessed practices of this review due to insufficient evidence; however, the overall strength of evidence is simply classified as suggestive due to the fact that most studies received a fair study quality rating. Despite there being no firm recommendation, the data do suggest that each of these three practices has the potential to improve times to initiate targeted therapy and possibly improve other patient outcomes.

The findings of effectiveness are based on three published and two unpublished studies for rapid molecular techniques without additional direct communication, four published and three unpublished studies on rapid molecular techniques with additional direct communication, and four published studies on rapid phenotypic techniques with additional direct communication. A number of the unpublished studies have since been published and are listed in the references. Of the 16 included studies, 2 were rated to be of poor quality and thus not used to determine recommendations; only 3 were rated as being of good quality for estimating the results most relevant to the review question, and 11 were rated as fair. Of the 14 studies of fair or good quality, most, 10 total, were judged to have substantial effect sizes for improving outcomes, while 3 were judged to have a moderate effect size, and 1 had an effect size ranked as minimal to none. For both practices involving rapid molecular techniques, the low strength of evidence is based on inconsistent findings (attributable to one study) and the overall lack of studies determined to be of good quality. Though the evidence was consistent for the practice of using phenotypic techniques with additional direct communication in improving outcomes, none of the four studies were rated as being of good quality and did not provide sufficient evidence supporting this practice.

The average standard difference in means for the three practices was as follows: −0.396 (95% CI, −0.888 to 0.44; not significant) for rapid molecular techniques; −1.483 (95% CI, −2.691 to −0.275; *P* < 0.05) for rapid molecular techniques, with additional direct communication; and −0.175 (95% CI, −0.279 to −0.072; *P* < 0.05) for phenotypic techniques, with additional direct communication. Standard differences in means less than zero favor the rapid test practice over the comparator practice. The meta-analysis results suggest that the implementation of any of the three practices may be more effective at increasing timeliness to targeted therapy than routine microbiology techniques for identification of the microorganisms causing BSIs. Based on the included studies, results for all three practices appear applicable across multiple microorganisms, including MRSA, S. aureus, Candida species, and Enterococcus species.

In conclusion, this article is a systematic review of the effectiveness of three rapid diagnostic practices for improving the timeliness of targeted therapy in patients with bloodstream infections (BSIs): rapid molecular techniques without additional direct communication, rapid molecular techniques with additional direct communication, and rapid phenotypic techniques with additional direct communication. The CDC-funded Laboratory Medicine Best Practices initiative systematic review methods for quality improvement practices were used. Fourteen studies met review inclusion criteria. Three were rated as being of good quality, and 11 were rated as fair. Most studies had substantial effect size ratings. The average standard difference in means for the three practices compared to more routinely performed practices favored the rapid test practice. However, because most studies were of only fair quality, the overall strength of evidence of effectiveness is only suggestive for each of the three practices in improving timeliness for targeted therapy in patients hospitalized with BSIs. Therefore, we are unable to make a recommendation for or against the three practices evaluated due to insufficient evidence. To create a more robust evidence base, a suggested roadmap for future studies is provided for use in preparing existing data or performing a prospective study for submission and effectiveness analysis for these practices (see Appendix 6).

## APPENDIX 1

### Elements Needed for Studies That Address This Systematic Review Question

1. General design. The study should be a pretest/posttest cohort study in defined hospital settings (i.e., large and small institutions) enrolling consecutive hospital inpatients suspected of having bloodstream infections (BSIs).
2. Sample size. The study sample should be sized according to the most relevant proximal outcome measure, i.e., the time to targeted therapy after conventional microbial identification techniques. We recommend that the study should be sized to detect ≥25% reduction in the time to targeted therapy, with a 95% CI. If possible, demographic information, such as those listed in the Standards for Reporting of Diagnostic Accuracy (STARD) recommendations (80), should be included to describe the patient population, including age, gender, description of hospital ward stratification, etc. (see Appendix 3). Diagnosis-related group information for the patients should be included as well. Microbial prevalence during intervention periods should also be reported, if available.
3. Practice (intervention) and comparator. Practices of interest include rapid molecular techniques and/or a variety of phenotypic techniques. As part of the practice, rapid and direct communication of results from these techniques to pharmacists or physicians is also of interest. Both pretest and posttest practices should be equally well articulated and recognizable from their descriptions. Information on how reporting and recording practices are performed should also be described.
4. Outcomes. Proximal, patient-centered outcome measures should include, for each patient, the time from the detection of the patient's BSI to the time that the patient was placed on targeted therapy, as well as the time required to report the identification of the microbe causing the BSI. Length of stay and mortality should also be measured. If possible, time points to be recorded include the following:
  a. the times that the BSI was detected and Gram stain results were reported to a clinician,
  b. the time that the patient was placed on initial therapy,
  c. the time that the identification and/or drug susceptibility test result was reported from the laboratory,
  d. the time that targeted therapy was confirmed or the patient was switched to targeted therapy, and
  the time that the pharmacist confirmed that the patient was on targeted therapy or the laboratory made a call to the clinician or infectious disease physician.
5. Analysis. Data analysis should include calculation of statistical significance of differences between the effects of the practice and comparator methods on the measured outcomes. Data averages (means) and standard deviations should be provided along with confidence intervals for all continuous data (e.g., time measures, including length of stay), while dichotomous measures (e.g., mortality) should include both the numerator (deaths) and denominator for both the pretest and posttest cohorts. These values will allow results to be included in the meta-analysis. If analyses linking rapid testing to distal morbidity and mortality outcomes are done, presentation upon admission and other patient characteristics and characteristics of their treatment should be statistically controlled in the analysis. Statistical assistance may be required to perform calculations.
6. Cost information. Cost information estimating all resources required for implementing and maintaining the intervention (new practice[s] of interest) and comparison practices, including, when appropriate, quantities and estimated unit costs and cost-related impacts, including savings, with supporting details associated with effectiveness outcomes are desired. Cost information observations should indicate the corresponding year and include reports concerning the following.
  a. What planning costs were incurred in preparing for the new testing technology and over what time period?
  b. What costs were incurred for purchasing new equipment, supplies, and software?
  c. What training costs, if any, were associated with instituting the new testing technology?
  d. What operational costs have been observed, and how do these observed costs compare with those of the alternative practice(s)?
  e. What operational and patient care-related savings have been observed?
  f. What additional financial consequences has your facility experienced as a result of instituting the new testing technology?
  g. Were costs normalized or adjusted to include the health care consumer price index?
7. Additional considerations. Additional observations about implementing the practice that are of interest include reports concerning the following.
  a. What training was required to institute the new testing technology?
  b. What systems modifications, if any, were required to accommodate implementation of the new practice?
  c. Provide detailed information on how batching of samples was handled and what staff hours and availability were for implementing both the practice and comparator techniques.

## APPENDIX 2

### Glossary

additional direct communication: An intervention communication by an infectious disease physician, pharmacist, or laboratory liaison recommending patient antimicrobial changes to the treating physician (51).
antimicrobial susceptibility testing: An *in vitro* laboratory test used to determine if an antimicrobial agent will be active in killing or inhibiting the growth of a specific microorganism (32).
batch testing: Laboratory tests performed based on designated time frames or when a specific number of tests has accumulated. This practice is chosen to save laboratory resources and reduce the cost per test (43).
bias: Systematic error, threats to validity, a tendency to produce results that depart systematically from the “true” results. Unbiased results are internally valid. There are many kinds of bias. Four common types of bias are selection/allocation, performance, measurement/detection, and attrition/exclusion.
blood culture: A set of two or more bottles of liquid culture medium into which a single blood specimen (typically 16 to 30 ml) is inoculated to detect the presence of bacteria in the blood. Normally, two bottles (one to two aerobic bottles and one anaerobic bottle or two aerobic bottles only) are inoculated for adults and one bottle for children (19, 32).
bloodstream infection: An infection associated with bacteremia or fungemia (32).
broad-spectrum antibiotic: An antibiotic that has the capacity to kill or inhibit two or more types of bacteria (19).
census: All eligible patients within a specified time period.
consistency: The degree to which estimates of effects (specific outcomes) are similar across the included studies.
continuous-monitoring blood culture systems: Laboratory instruments into which blood culture bottles containing the patient's blood are placed and monitored over the course of 24 h for 5 to 7 days in order to detect increased CO_2_ production or to measure gas pressure in the headspace of the culture bottle when microbial growth occurs (19, 32).
critical value: The first laboratory result (e.g., Gram stain or another rapid test) which is communicated verbally and/or electronically to a licensed health care provider as soon as possible (within 60 min) after laboratory verification of the abnormal result (19, 32). See the definition for “direct communication.”
direct communication: Active transfer of laboratory test results (of critical value) to a licensed caregiver by telephone or other direct electronic method rather than passive communication of information into the laboratory information system. Initial blood culture test results are routinely reported to a clinician's staff by the laboratory for the particular patient as soon as they are available.
*d*-score: Standardized difference in means where the mean from one group ($\overset{¯}{X}$*_G2_*) is subtracted from the mean from the second group ($\overset{¯}{X}$*_G1_*) and the result is divided by the pooled standard deviations of the means (*S_p_*). The *d*-score represents a position on a *z*-distribution.

$${ES}_{sm}=\frac{{\overset{¯}{X}}_{G1}-{\overset{¯}{X}}_{G2}}{S_{p}}$$

effect size: A value which reflects the magnitude of the difference between a study's outcome measures for the group in which the intervention/practice was evaluated and those of its control or comparison group.
empirical therapy: Initiation of broad-spectrum treatment before a firm diagnosis or etiology is determined (43).
external validity, generalizability, applicability: Extent to which the effects observed in the study are applicable to other populations and settings outside the study.
false negative: A culture bottle set that may not grow a microorganism for a few possible reasons, namely, (i) the infecting organism is not supported by the culture medium, (ii) the organism cannot be grown in culture and requires serological or molecular amplification, or (iii) the organism is inhibited by the presence of antimicrobial agents or human immune factors present in the blood prior to collection of the culture sample (19).
false positive: A blood culture contaminated with one or more organisms, typically skin flora. False-positive blood cultures are commonly contaminated with organisms introduced from the skin during specimen collection, typically coagulase-negative staphylococci, viridans group streptococci, corynebacteria, Propionibacterium spp., or Bacillus spp., not Bacillus anthracis. It is very important to rapidly determine whether Gram-positive cocci observed in clusters are coagulase-negative staphylococci or S. aureus and to further confirm whether the S. aureus is methicillin resistant (19).
fungemia: The presence of fungi (yeasts or molds) in the bloodstream (19, 32).
Gram stain: A differential stain used primarily to separate bacteria into two large groups (Gram positive and Gram negative) based on the physical properties of their cell walls. Bacteria can further be distinguished visually based on their shape, size, and arrangement. Gram stain characteristics may be used to classify bacteria into genera and determine or assist in switching from empirical to a more targeted therapy until more specific genus/species identification and susceptibility test results are available (43).
*I*^2^: A statistic related to *Q* which quantifies the proportion of total variability owing to heterogeneity. *I*^2^ is a standardized statistic, generally stated as a percentage between 0% and 100%.
internal validity: Extent to which the design and conduct of the study are likely to prevent systematic error. Internal validity is a prerequisite for external validity.
meta-analysis: The process of using statistical methods to standardize and quantitatively combine the results of similar studies in an attempt to allow inferences to be made from the sample of studies and be applied to the population of interest.
MicroScan system (Siemens): An automated phenotypic bacterial identification and drug susceptibility testing system. The system has two types of testing formats: one for conventional overnight testing and another for rapid testing (63).
molecular test: A diagnostic test that analyzes the presence or expression of genes or smaller genetic targets, DNA mutations, or RNA sequences. Examples of molecular tests are PCR and PNA-FISH.
odds ratio (OR): The ratio of the odds of an event in one group to the odds of an event in another group. One group is the treatment or intervention group and the other group is the control or usual-practice group. The odds in the treatment group are usually divided by the odds in the control or usual-practice group. An OR of 1 means the two practices are equally successful (no difference in reducing risk with respect to the outcome evaluated); an OR of >1 means the treatment or intervention practice is more successful; and an OR of <1 means the treatment or intervention practice is less successful. When the risk is small, odds ratios are very similar to risk ratios.
PCR: A molecular technique which amplifies a small amount of a microorganism DNA by logarithmic magnitudes so that it can be detected for diagnosis of pathogens and drug resistance (36, 37).
peptide nucleic acid fluorescent *in situ* hybridization (PNA-FISH) probes: DNA mimics with a noncharged peptide backbone. This allows the probes to target individual-species-specific targets on the 16S rRNA within bacteria or the 18S rRNA within fungi (54).
phenotypic test: Nonmolecular tests which measure activities such as enzymes or the presence of certain antigens, etc. (36, 37).
*Q*: By convention, a statistic which measures heterogeneity. The traditional *Q* is Cochran’s *Q*, which is a dimensional extension of McNemar’s test, the latter being a test for homogeneity in 2-by-2 tables. In the meta-analysis arena, *Q* tests for heterogeneity across some set of *n* different. A shortcoming of Cochran’s *Q* is its poor performance when *n* is small, which is frequently the case in meta-analysis. In response to this weakness, another *Q* statistic—Cohen’s *q*, or Cohen’s *Q*—has been defined by the statistical community and applied commonly in meta-analysis. It is a measure of heterogeneity which relies on correlations between the studies. Cochran’s *Q* has a χ^2^ distribution. Cohen’s *q* is standard normal.
rapid diagnostic technique: A microorganism identification or susceptibility test performed within the time frame of <6 to 8 h (although some tests are performed in time frames of 2 to 3 h), as defined by agreement of the BSI expert panel (see Appendix 3).
septicemia: A serious systemic illness caused by microorganisms and microbial toxins circulating in the bloodstream (52).
standardized: Transformed to a common metric allowing direct comparison of data across studies.
standardized difference in means (*d*-score): Difference in pretest and posttest means divided by the pooled standard deviation (see entry for *d*-score).
systematic review: A scientific investigation that focuses on a specific question and that uses explicit, planned scientific methods to identify, select, assess, and summarize the findings of similar but separate studies. It may or may not include a quantitative synthesis of the results from separate studies (meta-analysis).
targeted therapy: Antimicrobial treatment directed specifically to a microorganism based on the genus and species of that organism or actual susceptibility test results (30).
turnaround time (TAT): The time that it takes to perform and report a laboratory test result from the time that the sample is received in the laboratory.
transparency: Methods are explicitly defined, consistently applied, and available for public review so that observers can readily link judgments, decisions, or actions to the data on which they are based. Transparency allows users to assess the strengths and weaknesses of the systematic review and to provide associated guidance and recommendations.
Vitek system (bioMérieux): An automated antimicrobial system used for performing identification and drug susceptibility testing on microorganisms. The Vitek system uses a number of different card-based bacterium, yeast, and antibiotic susceptibility panels. The Vitek 1 system is for 18- to 24-h testing, while the Vitek 2 system can be used for 18- to 24-h testing as well as for rapid identification, for which results are generally completed within 6 to 8 h (36, 37).
weighted difference in means: The difference between two means weighted by the precision of the study.
without additional direct communication: The standard practice of communicating a critical value (an initial positive result) for a positive blood culture laboratory test result to a licensed caregiver, and when an additional rapid test result and/or culture result is available, the blood culture report is updated in the computer without direct communication to the caregiver.

## APPENDIX 3

### LMBP Bloodstream Infection Expert Panel Members

John Fontanesi, Ph.D.
Director, Center for Management Science in Health, and
Professor of Pediatrics and Family and Preventive Medicine
University of California, San Diego, CA
Lee Hilborne, M.D., M.P.H.
Professor of Pathology and Laboratory Medicine
Center of Patient Safety
David Geffen School of Medicine
UCLA, and
Quality & Corporate Medical Director
Quest Diagnostics
Ruth Pollison, M.S., M.T.(A.S.C.P.)
Laboratory Director
Newton Memorial Hospital
Newton, NJ
Michael Saubolle, Ph.D.
Medical Director
Microbiology and Department of Clinical Pathology
Banner Good Samaritan Medical Center
Banner Health, and
University of Arizona College of Medicine
Phoenix and Tucson, AZ
Melvin P. Weinstein, M.D.
Chief of the Division of Allergy, Immunology, and Infectious Disease
Rutgers Robert Wood Johnson Medical School
New Brunswick, NJ
David B. Wilson, Ph.D.
Professor and Chair of Criminology, Law, and Society
George Mason University
Fairfax, VA
Donna M. Wolk, Ph.D., D.(A.B.M.M.)
System Director of Clinical Microbiology
Geisinger Health System
Danville, PA, and
Professor
Wilkes University
Wilkes-Barre, PA

## APPENDIX 4

### LMBP Workgroup, 2011

Robert H. Christenson, Ph.D., D.A.B.C.C., F.A.C.B.
University of Maryland Medical Center
Baltimore, MD
John Fontanesi, Ph.D.
UC—San Diego Medical School
San Diego, CA
Julie Gayken, M.T.(A.S.C.P.)
HealthPartners Medical Group Clinics, and
Regions Hospital
Bloomington, MN
James Nichols, Ph.D.
Vanderbilt University Medical Center
Nashville, TN
Mary Nix, M.S., M.T.(A.S.C.P.), S.B.B.
Agency for Healthcare Research and Quality
Rockville, MD
Sousan S. Altaie, Ph.D. (*ex officio*)
Food and Drug Administration
Silver Spring, MD
Melissa Singer (*ex officio*)
Centers for Medicare and Medicaid Services
Baltimore, MD
Raj Behal, M.D., M.P.H.
Rush University Medical Center
Chicago, IL
Cyril (Kim) Hetsko, M.D., M.P.H.
Trustee
American Medical Association
Madison, WI
Lee Hilborne, M.D., M.P.H.
David Geffen School of Medicine,
UCLA, and
Quest Diagnostics
Los Angeles, CA
Stephen Raab, M.D.
Memorial University St. John's, Newfoundland, Canada, and
Clinical Chief of Laboratory Medicine, Eastern Health Authority
Newfoundland, Canada
Milenko Tanasijevic, M.D., M.B.A.
Brigham and Women's Hospital
Boston, MA
Ann M. Vannier, M.D.
Southern California Kaiser Permanente
Regional Reference Laboratory
North Hollywood, CA

## APPENDIX 5

### Evidence Summary Tables for Rapid Molecular Techniques with No Additional Direct Communication Practice

Consult Tables A1 to A7 for evidence summary tables for rapid molecular techniques with no additional direct communication. For scoring information, see Christenson et al. (1). Boldface results were used for analysis. Papers with outcome measures of interest (but not those outcomes considered relevant for analysis purposes) are also included. No effect rating or relevance is provided for those studies.

**TABLE A1:**
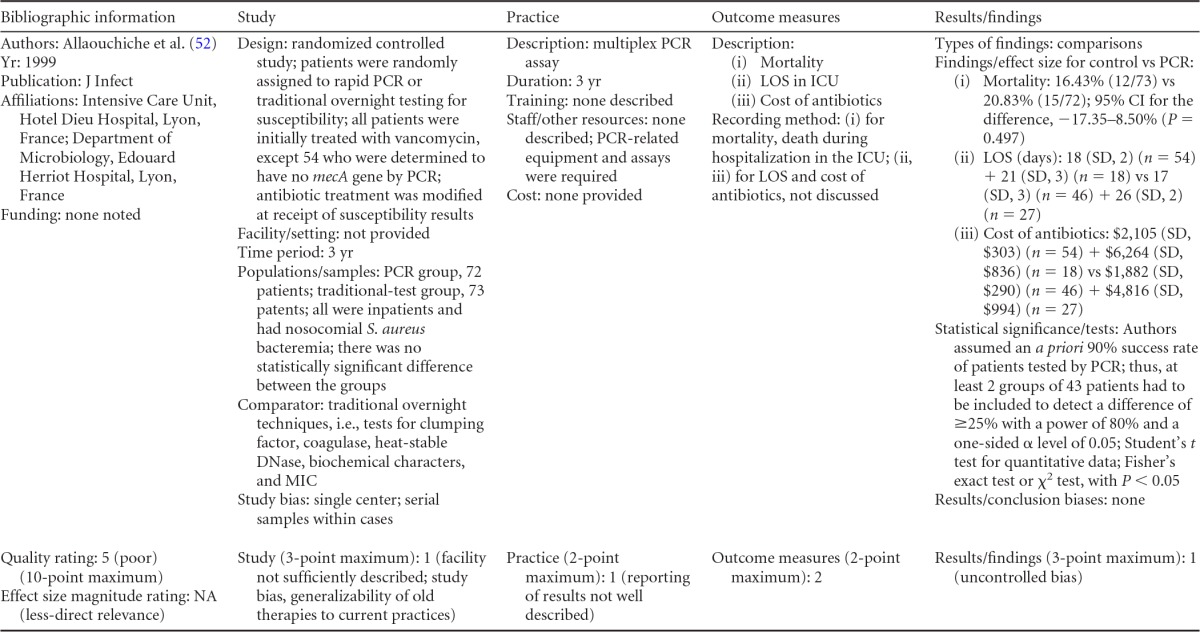
Evidence summary table and scoring criteria for reference 52^*a*^

a Roman numerals within and between columns refer to the descriptions under “Outcome measures.” NA, not applicable.

**TABLE A2:**
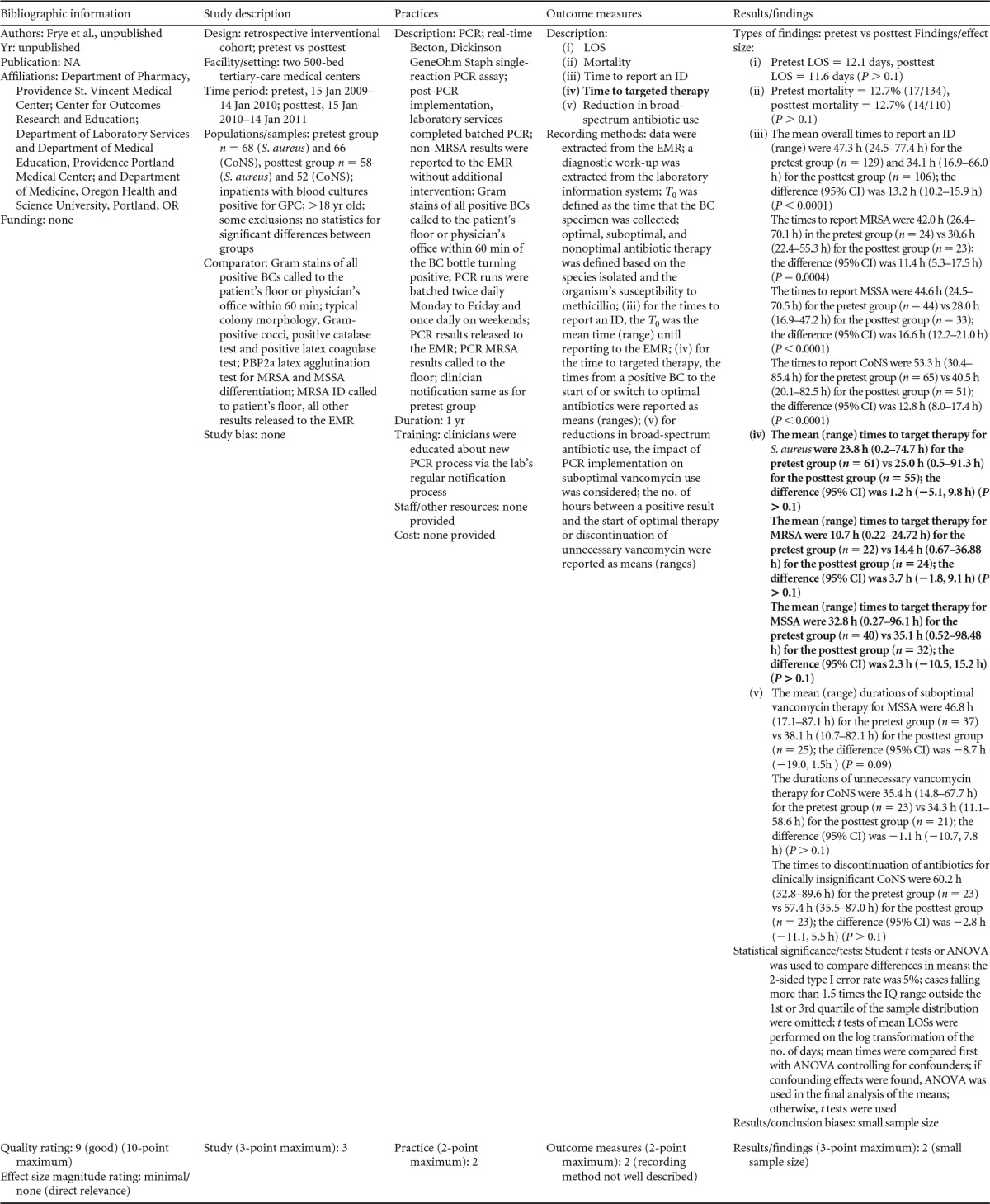
Evidence summary table and scoring criteria for Frye et al., unpublished (now published as reference 51)^*a*^

a EMR, electronic medical records; *T*_0_, time zero; ANOVA, analysis of variance; IQ, interquartile range.

**TABLE A3:**
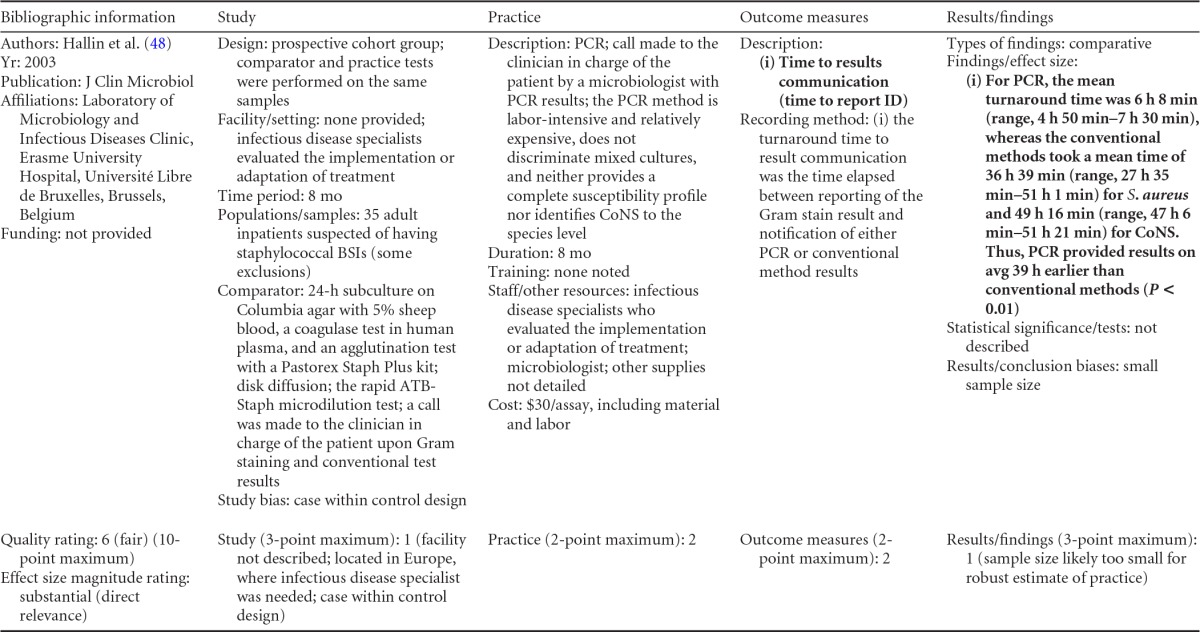
Evidence summary table and scoring criteria for reference 48

**TABLE A4:**
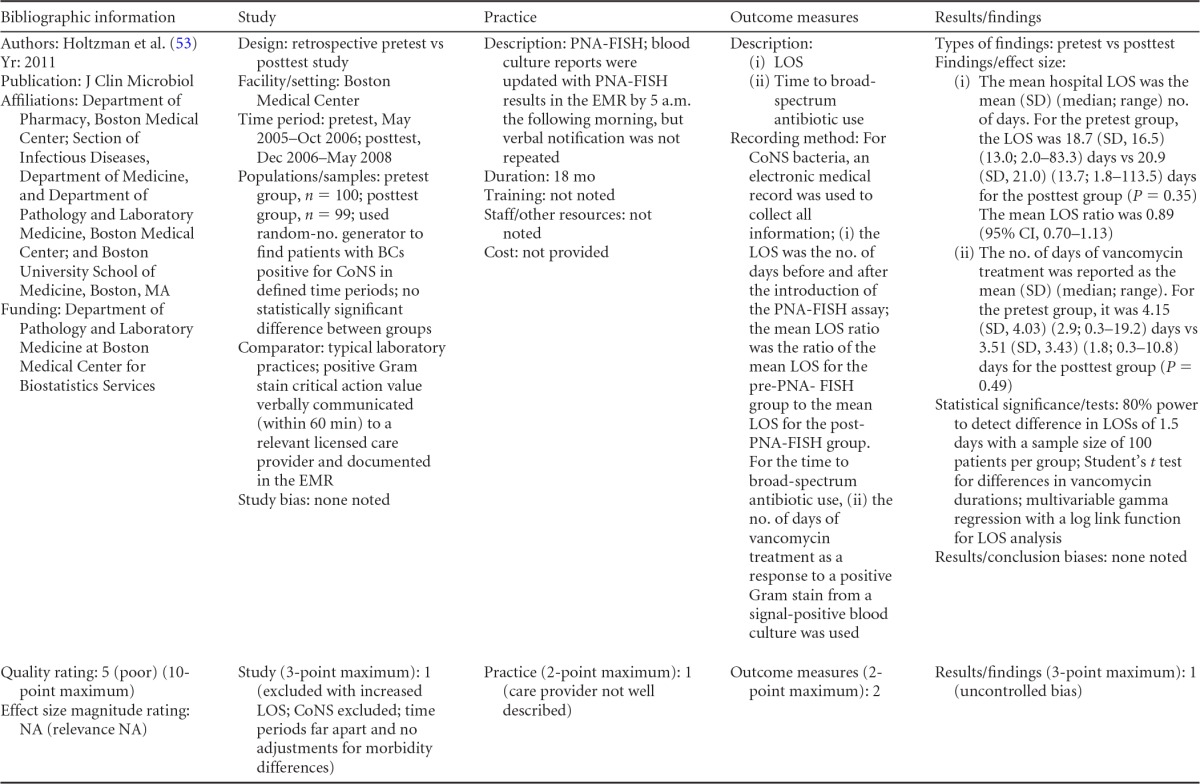
Evidence summary table and scoring criteria for reference 53

**TABLE A5:**
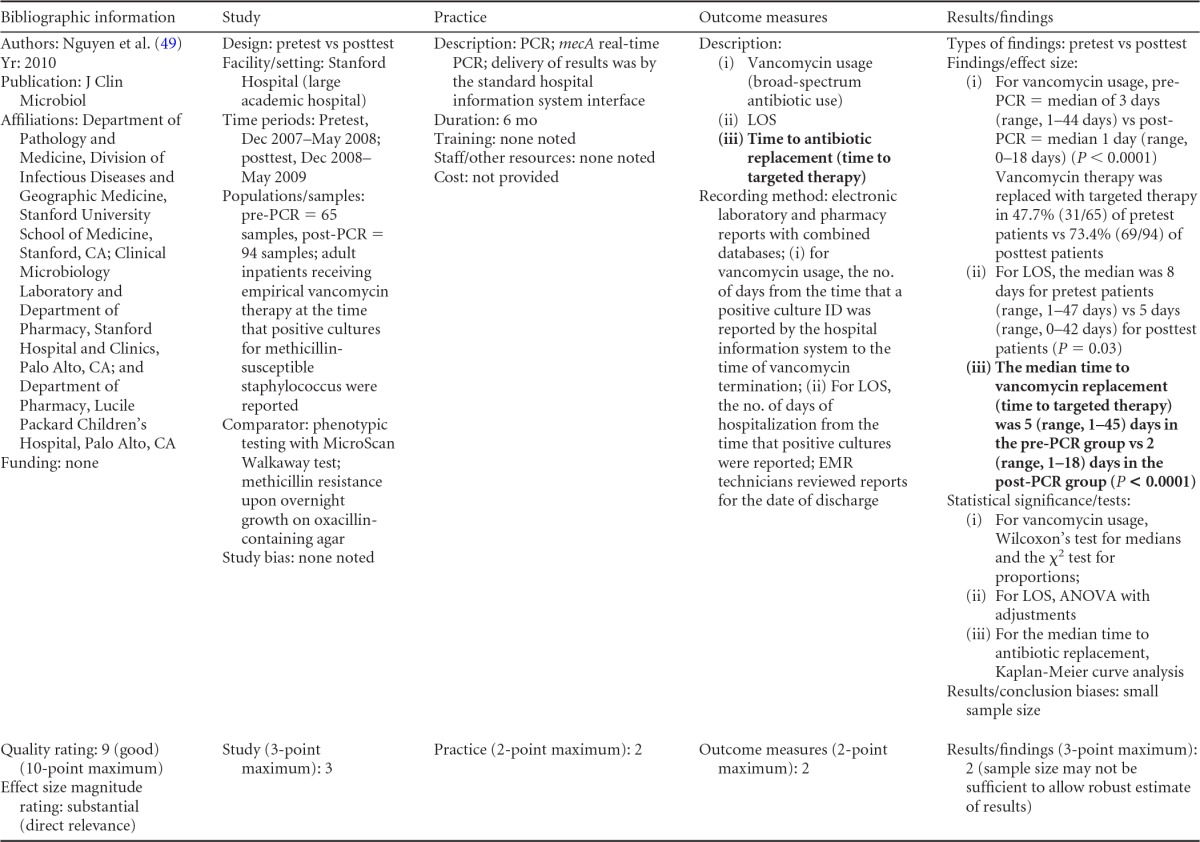
Evidence summary table and scoring criteria for reference 49

**TABLE A6:**
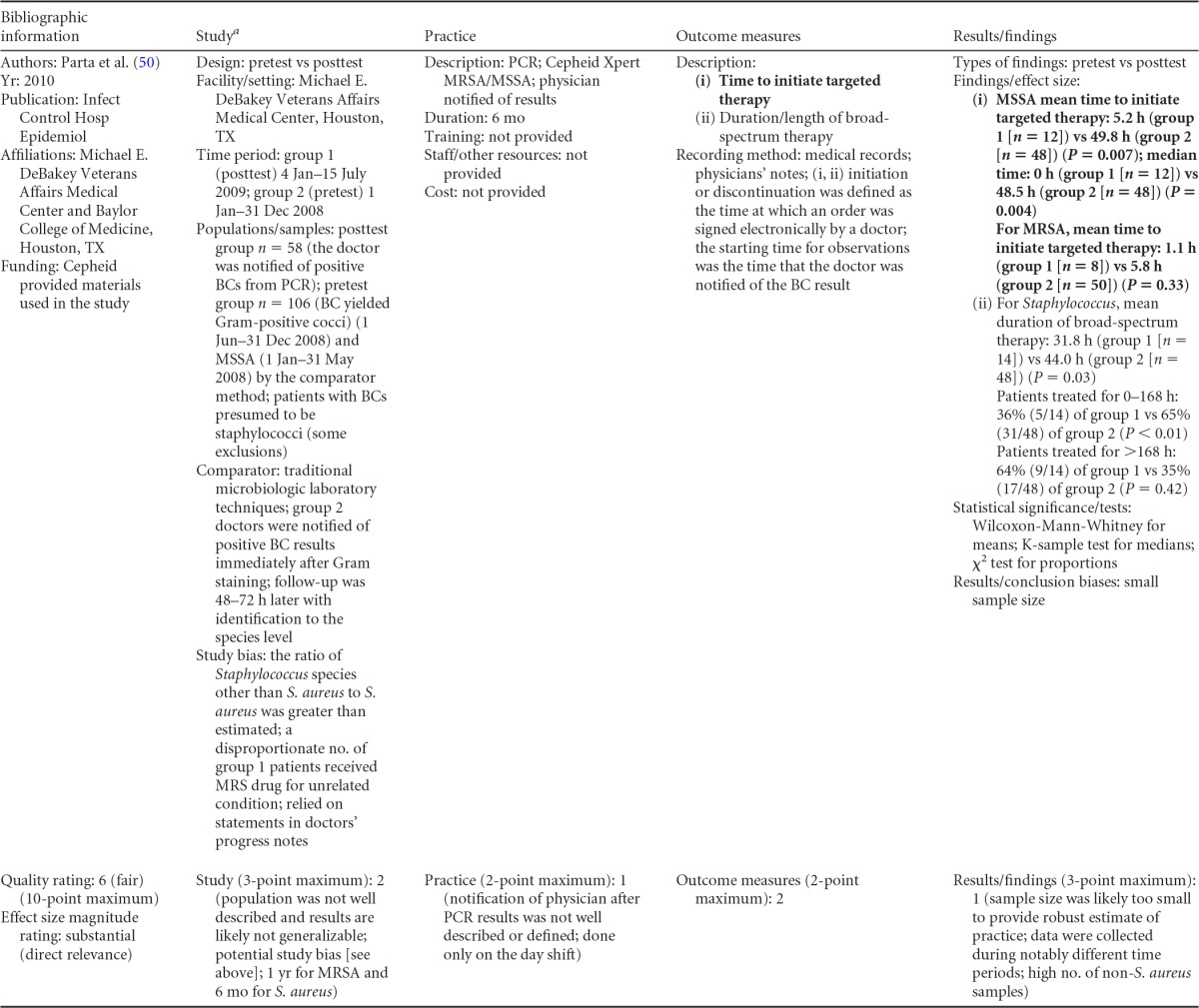
Evidence summary table and scoring criteria for reference 50

a MRS, multidrug-resistant *Staphylococcus*.

**TABLE A7:**
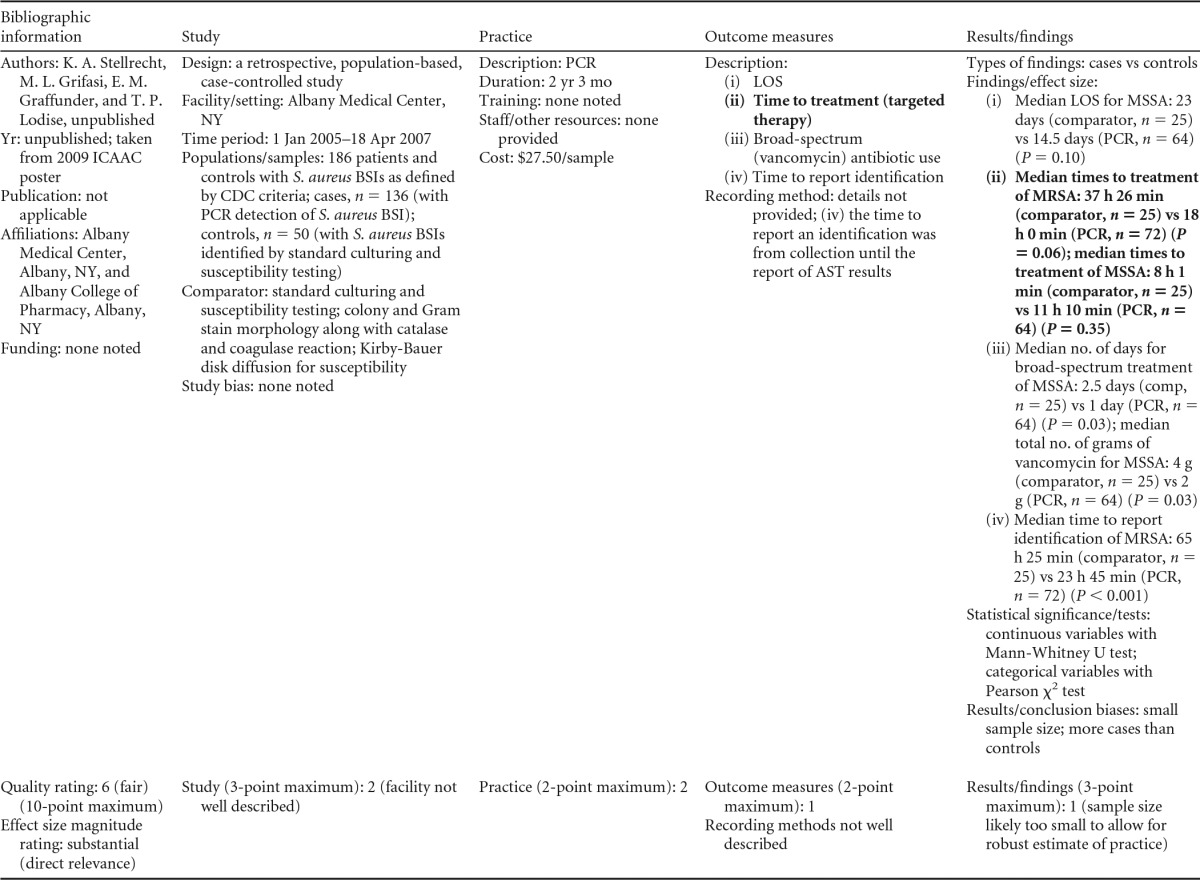
Evidence summary table and scoring criteria for unpublished data contributed by listed authors

### Evidence Summary Tables for Rapid Molecular Techniques with Additional Direct Communication Practice

Tables A8 to A19 are evidence summary tables for rapid molecular techniques with additional direct communication. For scoring information, see Christenson et al. (1). Boldface results were used for analysis. Papers with outcome measures of interest (but not those outcomes considered relevant for analysis purposes) are also included. No effect rating or relevance is provided for those studies.

**TABLE A8:**
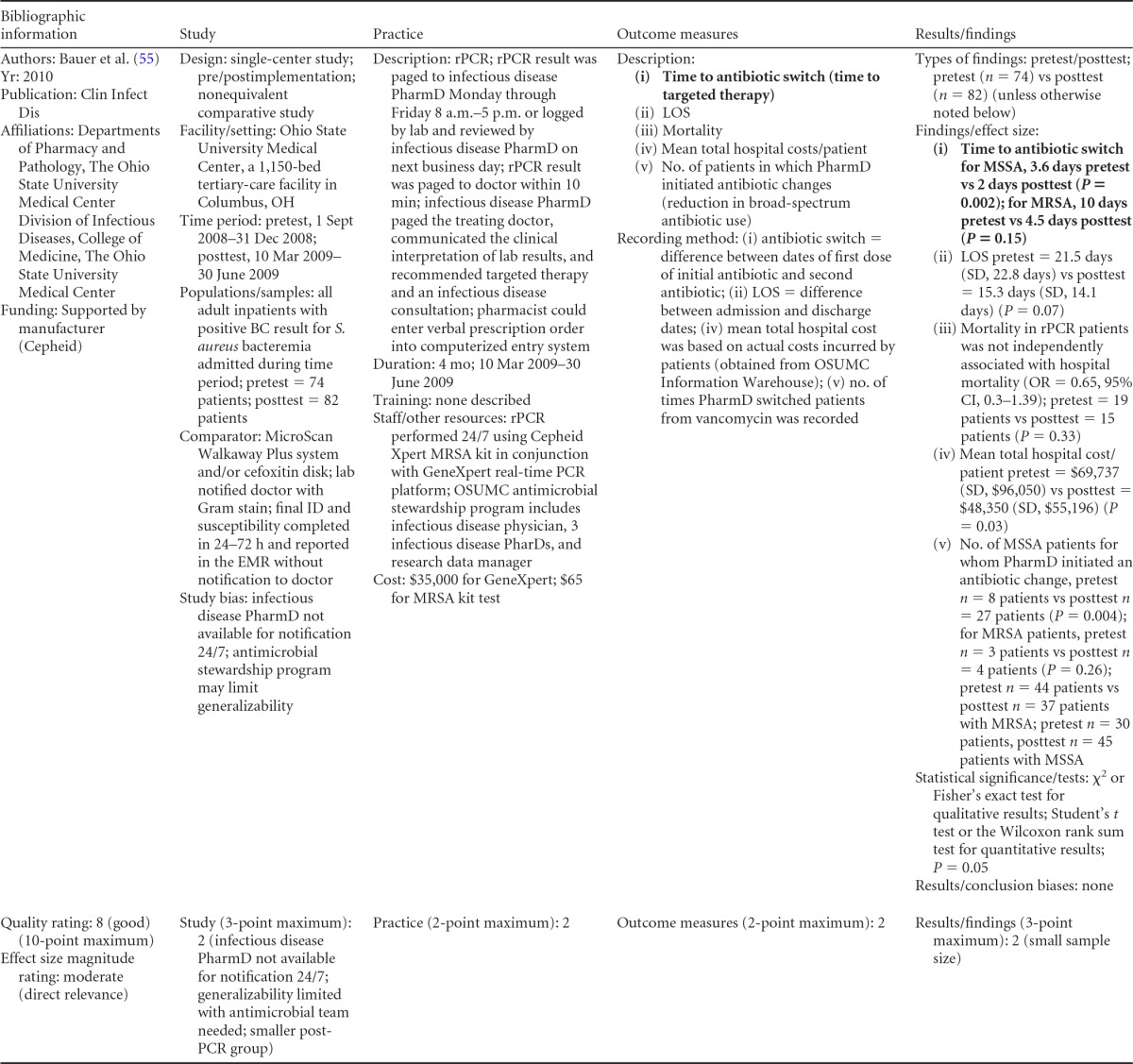
Summary table and scoring criteria for reference 55^*a*^

a ID, identification; EMR, electronic medical record; PharmD, doctor of pharmacy; rPCR, rapid PCR.

**TABLE A9:**
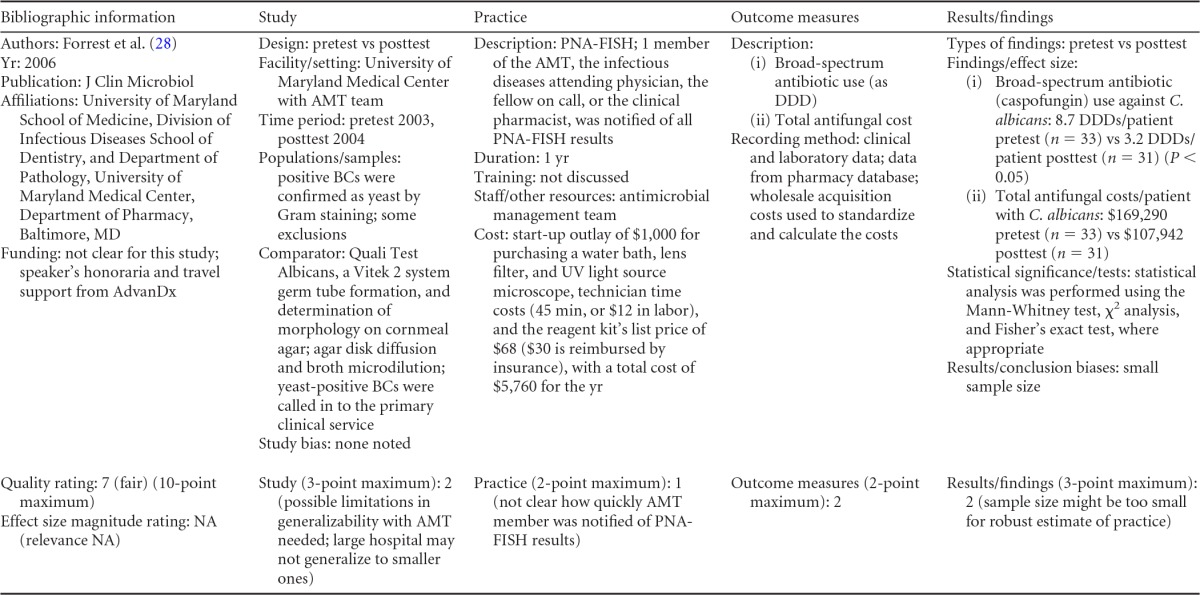
Evidence and summary table and scoring criteria for reference 28^*a*^

a AMT, antimicrobial management team; DDD, defined daily dose.

**TABLE A10:**
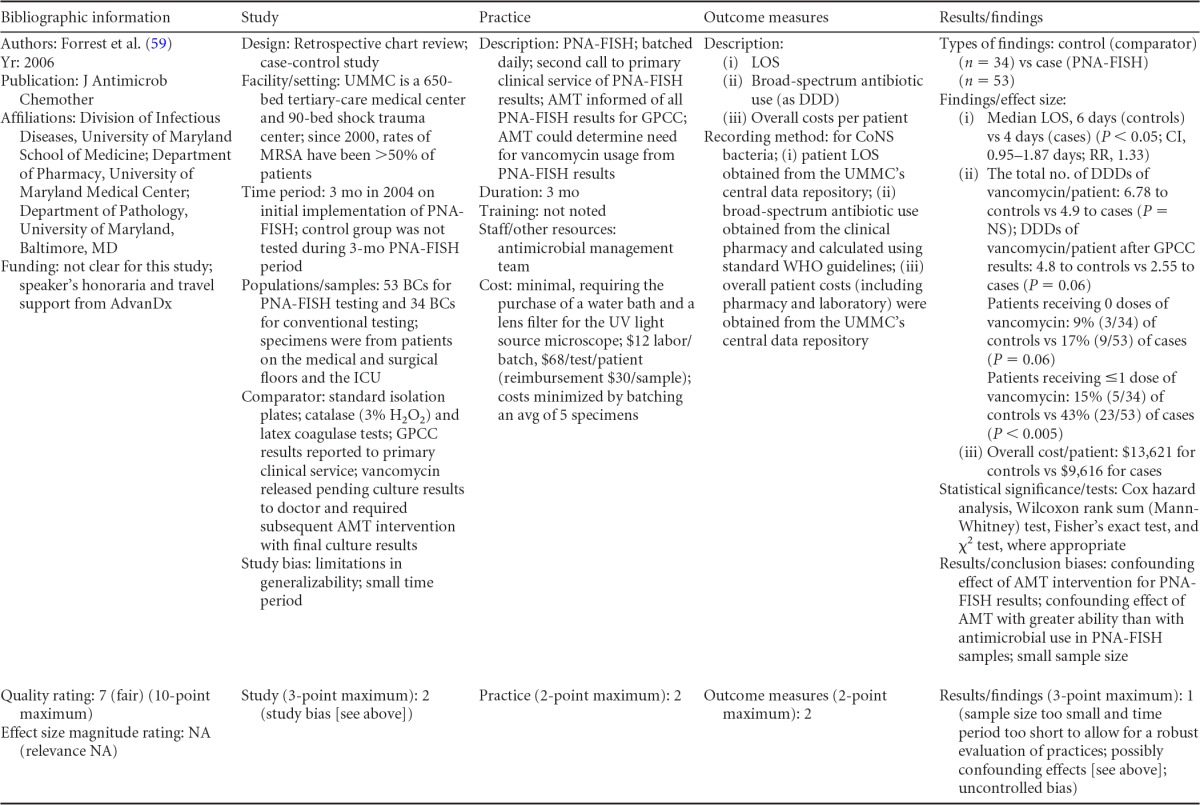
Evidence summary table and scoring criteria for reference 59^*a*^

a RR, relative risk; NS, not significant; DDD, defined daily dose.

**TABLE A11:**
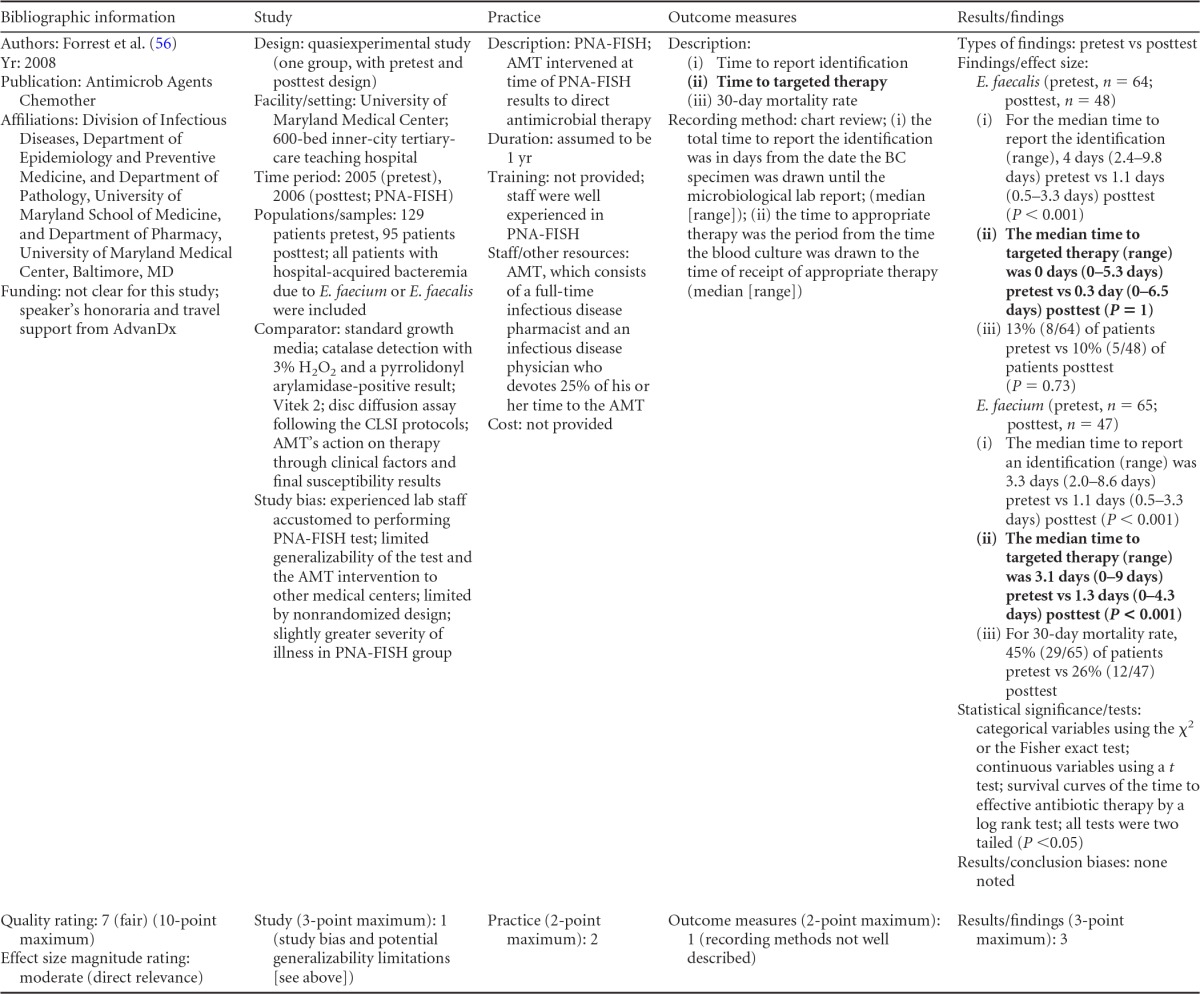
Evidence summary table and scoring criteria for reference 56^*a*^

a AMT, antimicrobial management team.

**TABLE A12:**
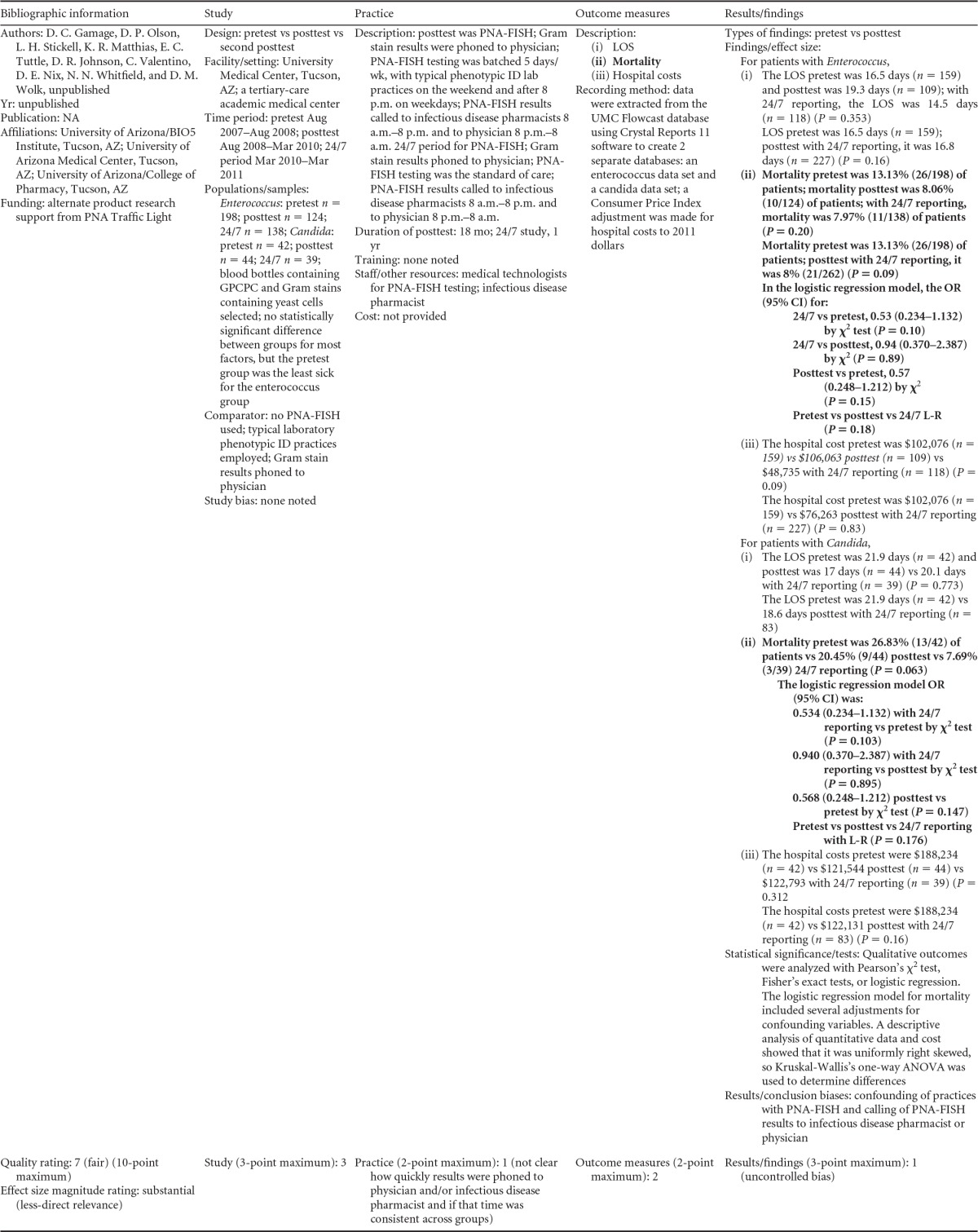
Evidence summary table and scoring criteria for unpublished data contributed by authors listed^*a*^

a GPCPC, Gram-positive cocci in pairs and chains; L-R, logistic regression.

**TABLE A13:**
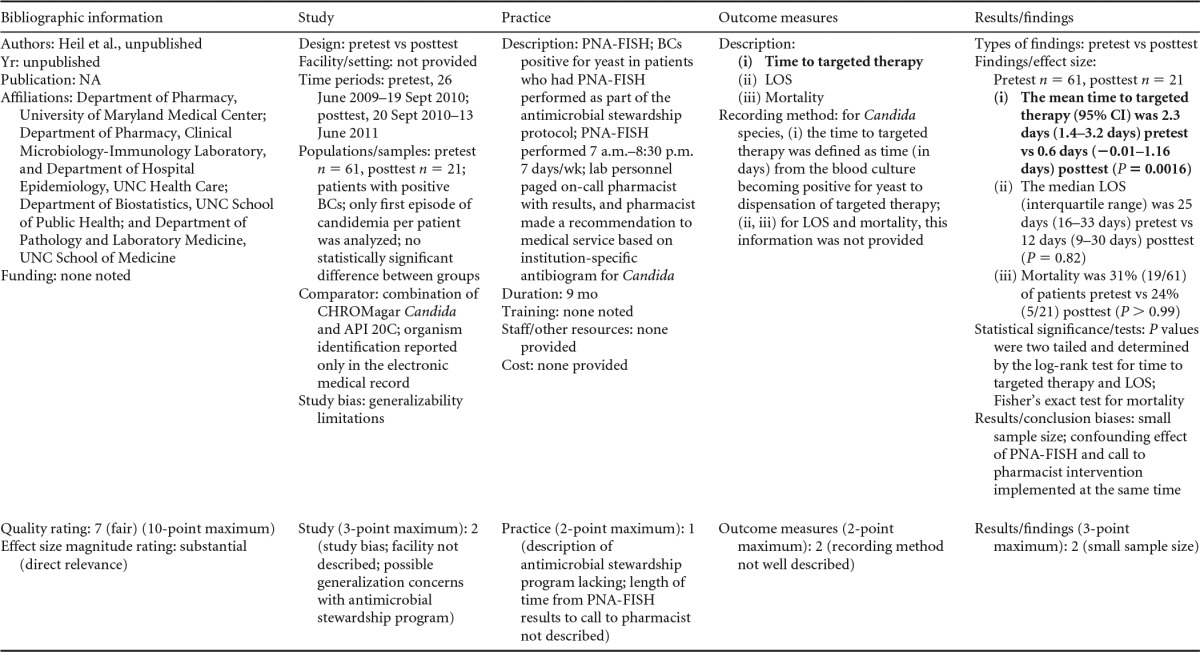
Evidence summary table and scoring criteria for unpublished data contributed by authors listed (now published as reference 58)

a LCL, laboratory/clinician liaison; GPCC, Gram-positive cocci in clusters; LIS, laboratory information system.

**TABLE A14:**
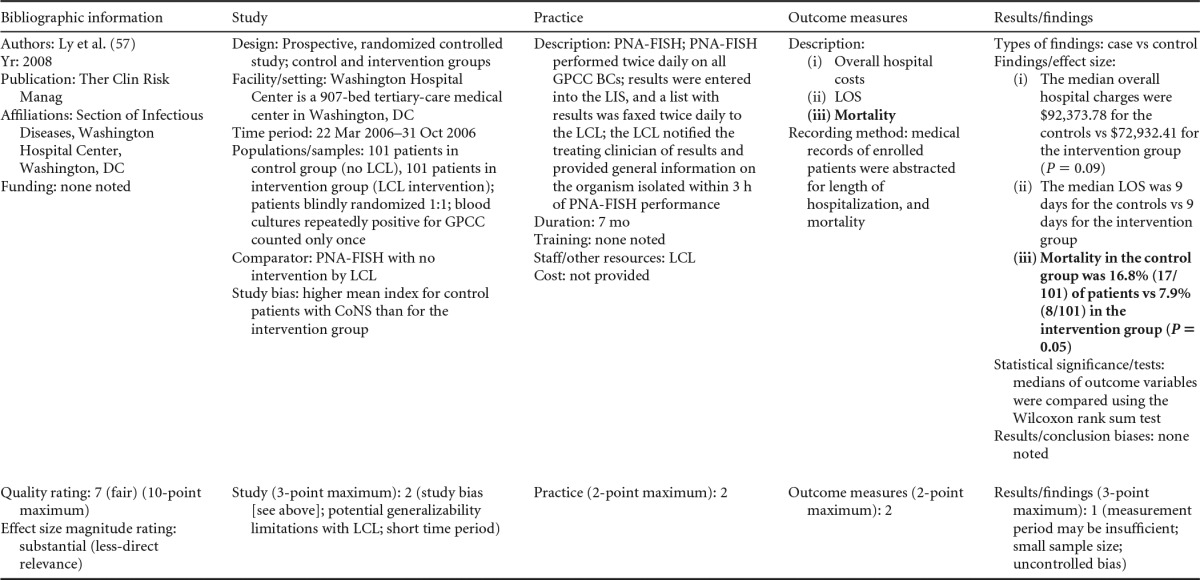
Evidence summary table and scoring criteria for reference 57^*a*^

a LCL, laboratory/clinician liaison; GPCC, Gram-positive cocci in clusters; LIS, laboratory information system.

**TABLE A15:**
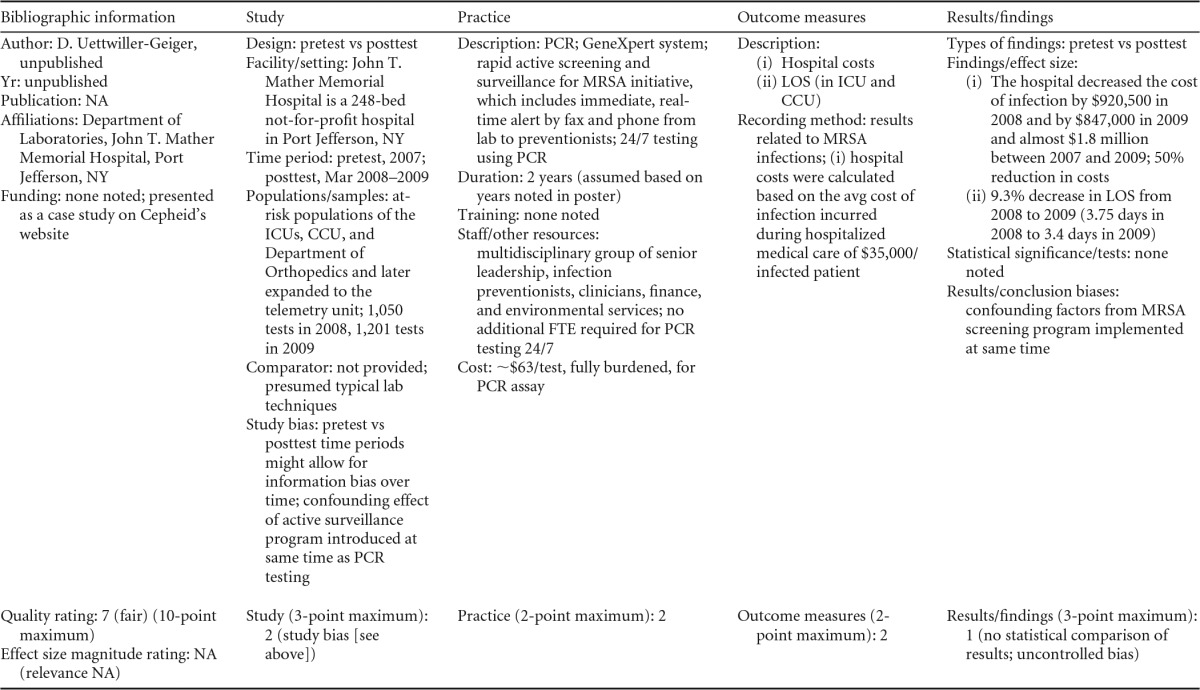
Evidence summary table and scoring criteria for unpublished data contributed by author listed^*a*^

a ICUs, intensive care units; CCU, cardiac care unit; FTE, full-time employee.

**TABLE A16:**
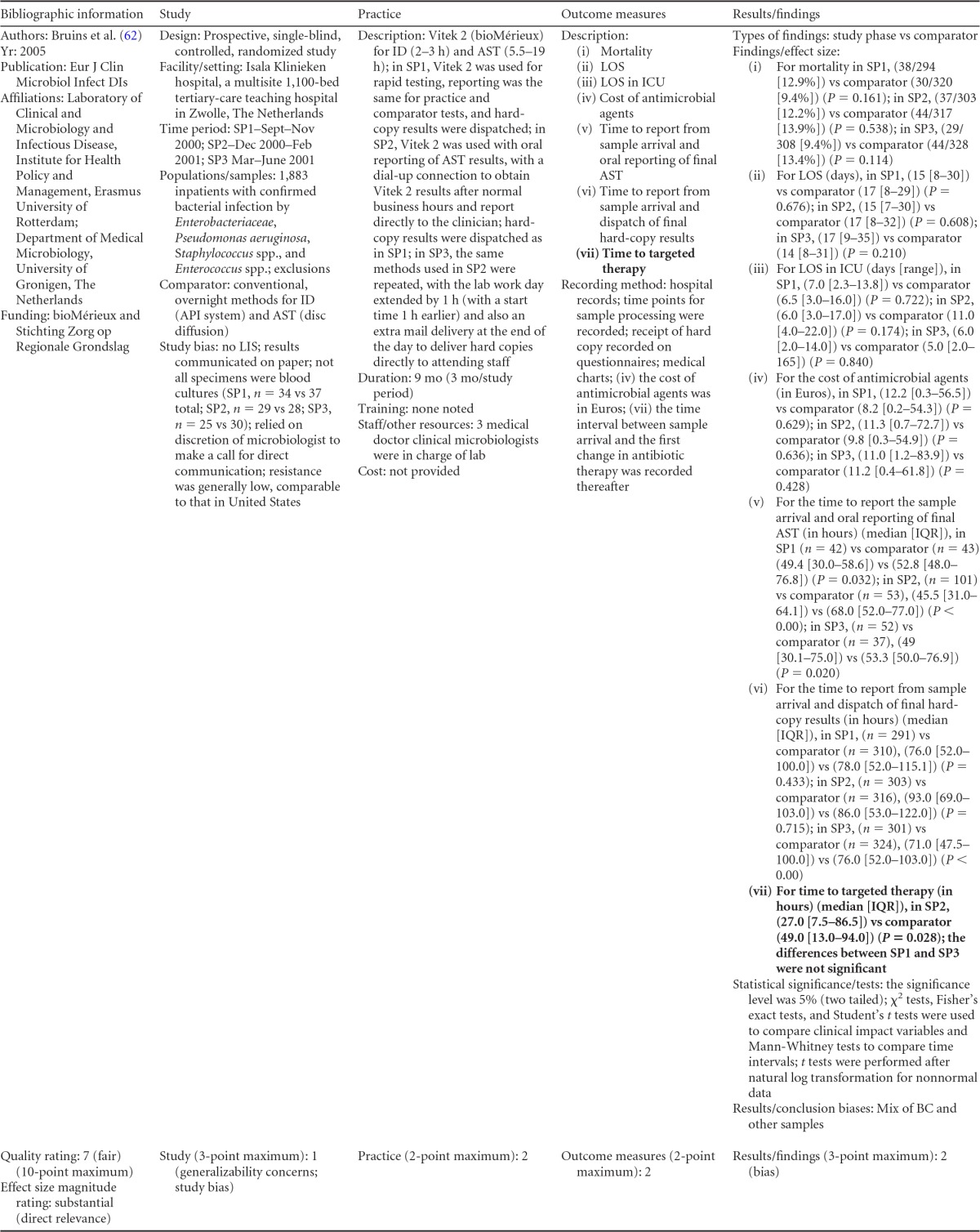
Evidence summary table and scoring criteria for reference 62^*a*^

a LIS, laboratory information system; SP1, SP2, and SP3, study phases 1, 2, and 3, respectively.

**TABLE A17:**
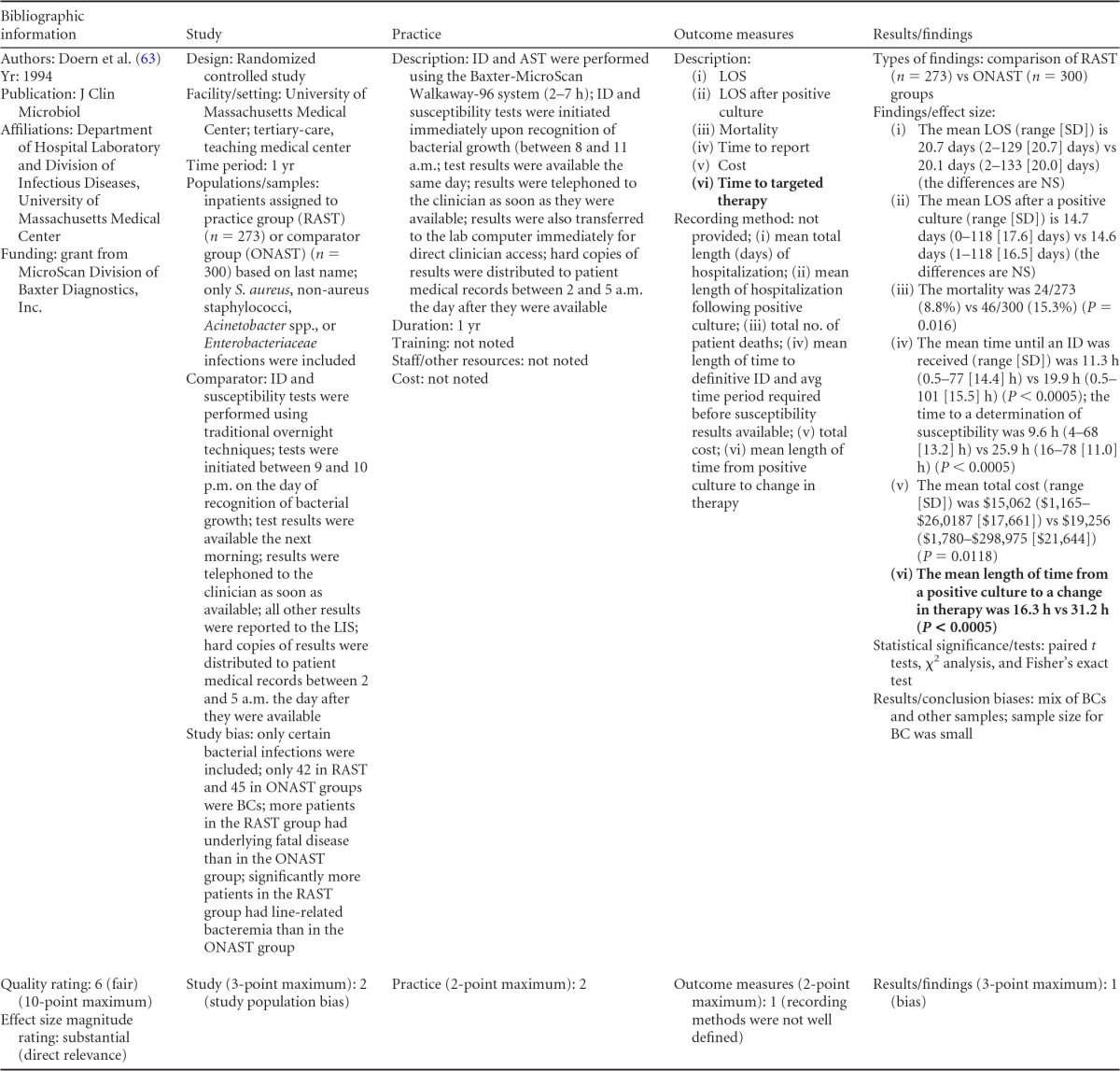
Evidence summary table and scoring criteria for reference 63^*a*^

a LIS, laboratory information system.

**TABLE A18:**
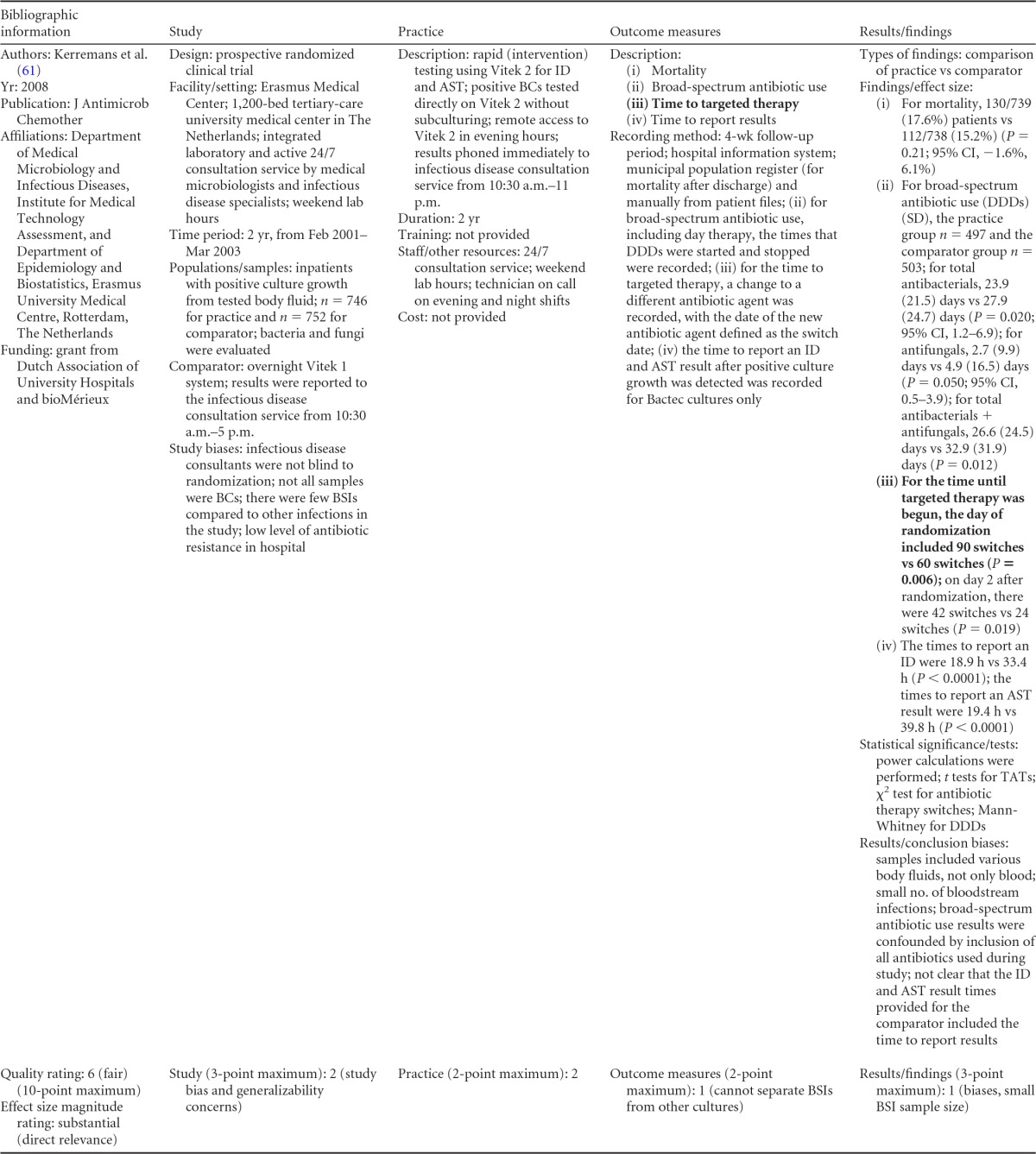
Evidence summary table and scoring criteria for reference 61^*a*^

a DDD, defined daily doses; TATs, turnaround times.

**TABLE A19:**
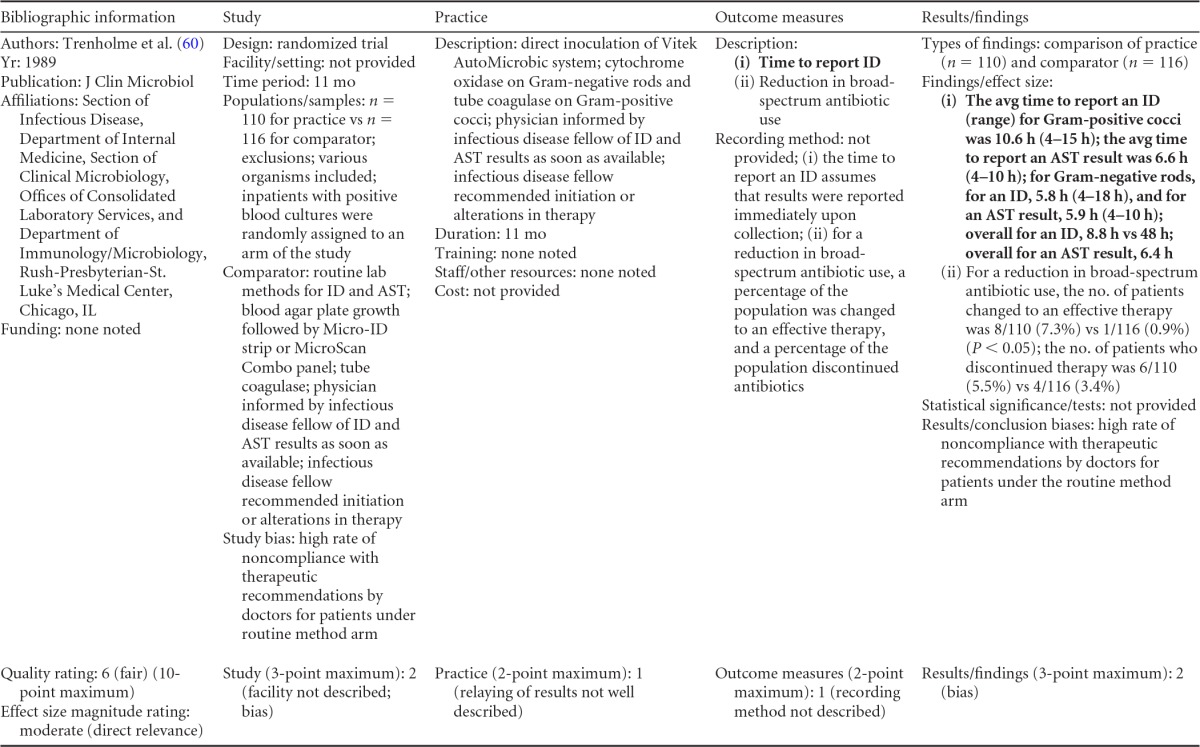
Evidence summary table and scoring criteria for reference 60

## APPENDIX 6

### LMBP Evaluation of Rapid Methods for Bloodstream Infection Identification: Suggested Roadmap for Future Quality Improvement Studies

Analysis of quality improvement study data for the purpose of a systematic review of evidence about laboratory practice effectiveness requires that some basic and essential parameters of study design be included, documented, and adequately described by laboratory investigators for more standard reporting and comparison (meta-analysis). This document is developed as a roadmap to organize, plan, and report a quality improvement project to address the following questions. For hospital inpatients with bloodstream infections (e.g., positive blood cultures), what practices are effective at increasing the timeliness of providing targeted therapy, and do these practices improve the patient's clinical outcome?

In general, the design, description, methods, data collection, and analysis for the study should be written and documented for replication by other laboratory investigators, with provision of results to verify or refute the original study. The following roadmap may be a useful outline for a study of this topic as well as guide data collection for a quality improvement project responsive to the evaluated practices of this review.

**FIG A1:**
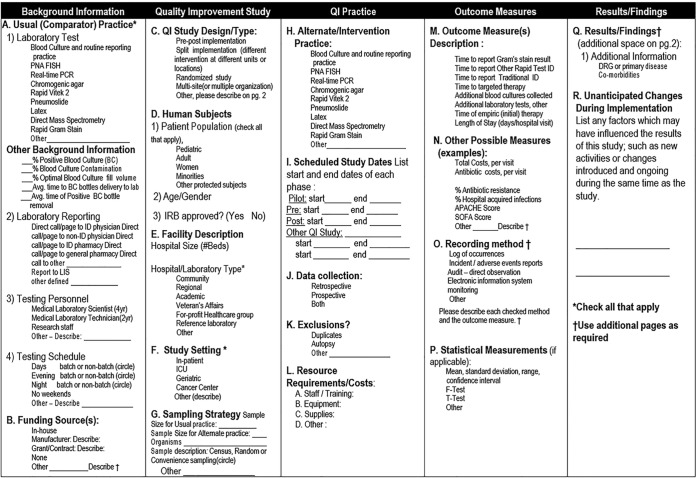
Chart to assist in planning future QI studies.

**FIG A2:**
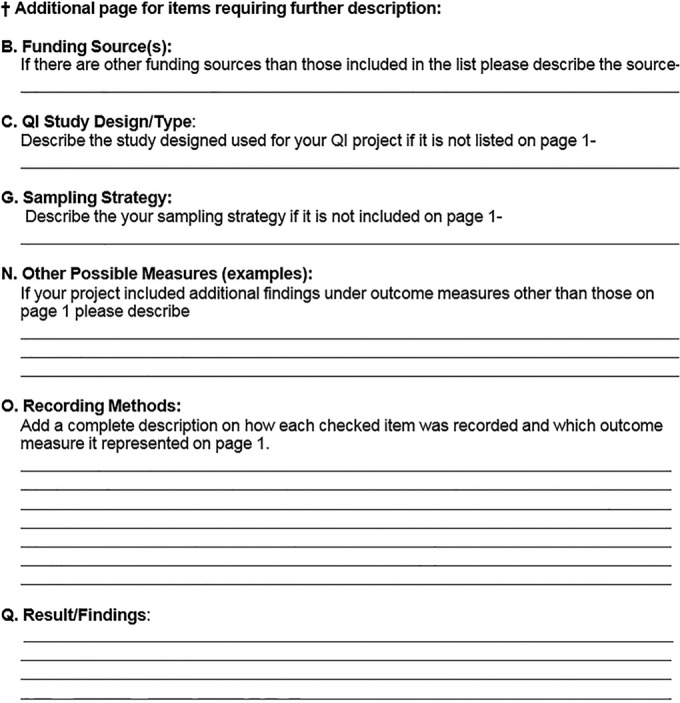
Second page of chart in Fig. A1 for items requiring further description.

Figures A1 and A2 show a data collection form including the criteria to consider and address when planning, conducting, and reporting this type of QI study. It may assist in addressing the questions to be asked and in defining the population, intervention, comparison, and outcome (PICO) elements that should be included as part of an evidenced-based QI study.

### Background Information

A. Usual (comparison) practices. This roadmap provides examples of traditional, rapid, or phenotypic identification techniques for BSIs and other laboratory practices, such as reporting, classification of testing personnel, and testing schedule, that are currently performed at your facility. Use the roadmap to select which of these elements your facility is currently following to identify and report bloodstream infections for inpatients.
  1. Laboratory test. In this checklist, identify the usual testing method(s) used by your facility. If your testing method is not listed, please add it in the Other space. Note that if your lab is currently using blood cultures for BSI testing, please indicate (if the data are available) the percentage of positive blood cultures, the percentage of contaminated blood cultures, and the percentage of bottles with optimal fill volume. Also, include the average time from the collection of blood cultures to arrival in the laboratory and the average time from when positive blood cultures are removed from the instrument to when Gram staining is performed.
  2. Laboratory reporting. Currently, how does your facility communicate positive bloodstream reports for inpatients? If the reporting protocol requires a direct call/page to the identified physician as well as reporting to the laboratory information systems (LIS), check both boxes. Additional spaces are provided if another practitioner is called or another protocol is followed.
  3. Testing personnel. Check which testing personnel are involved in completion of the patient testing at your facility. If a laboratory assistant preforms the preliminary lab set-up, such as preparation of Gram stain, but the reading and reporting of the test is conducted by the medical technologist please indicate the personnel for the final assessment of the test.
  4. Testing schedule. Indicate your current schedule for testing bloodstream specimens. If testing is conducted on a 24-h schedule, please circle all options that apply (days, evening, and night). Also, indicate the frequency of testing by circling “batch” or “non-batch.”
B. Funding source(s) (if applicable). List who may have assisted you financially in this quality improvement project for purposes of reporting in the final published account.

### Quality Improvement Study

C. Quality improvement study design/type. For planning purposes, select the quality improvement study type. For the BSI study, a pre/postimplementation design may easily capture the data needed for analysis. In this section, describe the methods/approaches that your facility will use. To limit bias (see the glossary in Appendix 2 and below), use similar patient population characteristics (age range, sex, ethnicity, and diagnosis). Pre/postimplementation measures outcomes of the usual testing practice (preimplementation) and measures outcomes after implementation of the alternate (intervention) practice (postimplementation). “Split implementation” means implementation of different interventions at different locations within the same institution. Your study may be a randomized, controlled study. A multisite trial (multiple facilities) consists of a coordinated series of trials across multiple institutions; i.e., the same research protocol is implemented in several sites. Studies of other formats should be described. Bias is the tendency to produce results that depart systematically from the “true” results. Bias is any nonrandom factor in the conduct of a study that can influence the results of a study. Four types of bias found in QI projects are selection/allocation, performance, measurement/detection, and attrition/exclusion (see http://bmg.cochrane.org/assessing-risk-bias-included-studies). Selection bias refers to systematic differences between baseline characteristics of the groups that are compared. For example, emergency room (ER) patients are compared to surgical patients. The bias occurs when there are systematic differences in patient characteristics which are associated with group assignment. Performance bias refers to systematic differences between groups in the care that is provided or in exposure to factors other than the interventions of interest. This may include services other than the practice being tested that are provided to one group but not the other. Exclusion bias occurs when you have an exclusive sample but claim that the results have relevance for a more general population. Measurement bias refers to systematic differences between groups in how outcomes are determined. Changes in how outcomes are measured or recorded may change the sensitivity of the measure for detecting an outcome. If so, the study may document changes in outcome which reflect the sensitivity of the measure and not the true rate of occurrence.
D. Human subjects.
  1. Patient population. Give as much information as possible about the group(s) you are selecting for the quality improvement study. Note that if there are any differences in patient mixes between those tested using the comparison practice and those tested using the alternative/intervention practice, bias may be introduced (see the discussion of selection/allocation bias above).
  2. The age and gender of each subject should be recorded on the study intake form and reported as the age range and the percentages of male and female subjects.
  3. IRB approval. Institutional review board (IRB) approval (or exemption) must be sought for any study before it has begun. Please consult with your relevant IRB official for appropriate procedures before beginning any study. LMBP welcomes the submission of unpublished QI data when the project is completed for inclusion in the next systematic review cycle (see http://wwwn.cdc.gov/futurelabmedicine/about/default.aspx).
E. Facility description. Hospital size is the number of beds. For the type of facility, place a checkmark beside the type of hospital or private/reference laboratory where the QI study will be conducted.
F. Study setting. Describe the setting(s) that will provide the patients or samples included in the study. For example, place a checkmark beside “ICU” (intensive care unit) if it will be the only nursing unit selected. If all hospitalized patients identified with a bloodstream infection will provide a larger sample, “Inpatient” would be the setting selected for the study. If your facility is designated a geriatric or cancer center but you select all hospitalized patients, then place a checkmark beside both.
G. Sampling strategy for BSIs. The sample size is the number of patients/observations used for the usual (comparison) practice and the alternate (intervention) practice. Use the largest available sample at each time of measurement. For results to be reliable, the implemented practice should be the only difference in the study populations influencing the results. Describe your sample (tests, patient specimens, patients, or type of patient specimens) and the sample size. An example is 15,000 patient specimens tested for the usual (comparison) practice (prepractice) and 13,200 patient specimens tested for the alternate (intervention) practice (postpractice). In addition, list the organisms selected/included in the study. Note that a power analysis should be performed by a statistician prior to confirmation of the sample size. Statistical power is the probability of concluding that there is a difference when there is, in fact, a difference between your standard method and your new method (i.e., the probability that your study will detect a difference, given that one truly exists. An example of a nomogram for sample size calculation may be viewed in reference 81. Indicate the sampling category. Census sampling includes all eligible patients within a specified time period. Simple random sampling includes subjects (patients) selected for study inclusion by a formal random selection process in which each patient in the census is equally likely to be selected for participation in the study. Convenience sampling includes some subset of the census population because it is easy to access. For example, using data only from patient records that you can easily reach would be a convenience sample. For the “other” category, describe whether you are using a different sampling method. If you are using anything other than a census, simple random, or convenience sampling, you will likely need a sampling statistician to construct an appropriate sampling strategy.

### QI Practice

H. Alternate/intervention practice. Describe the testing practice that you will implement as an alternative (intervention) to the comparison (current) practice (described in section A1). Some examples include rapid molecular techniques (real-time PCR, PNA-FISH) and/or a variety of phenotypic techniques.
I. Study dates. Prior to beginning the quality improvement study, it is recommended that you select dates for each phase of the study. A pilot period for evaluating the implementation of a new practice and confirming its full implementation is often advisable. Assessing the effectiveness of a practice will be done once it is fully implemented and functioning as intended. Another consideration for study dates would be to inquire about other changes that may impact your data collection schedule or confound the results of your QI study. For example, it would be best to avoid times scheduled for a system-wide computer upgrade or other large-scale projects that might impact measurement or otherwise create bias. If a pilot phase is done prior to your QI project, indicate the start and end dates. If a pre/postimplementation study is selected as your QI design, indicate the start and end dates of each phase. Any other QI study design (see section C [QI study/design type]) selected by your facility may require multiple dates for a measurement of the practices included in the project. Additional information of project dates may be included on a separate page.
J. Data collection. Are data collected as the study is being conducted (prospective), retrospectively (data taken from medical record charts), or both ways?
K. Exclusions. List any data that are excluded from the study, such as duplicates or autopsy or other samples.
L. Resource requirements/costs. Additional requirements and costs which are required to implement the practice should be listed. A full description of these adoptions and maintenance considerations should be included in the study write-up.

### Outcome Measures

M. Description of outcome measures. Options for outcome measures for your quality improvement study are listed below. A selection concerning the outcome measures should be finalized prior to the start of the study to ensure that the results will contribute to improved practices. Note the time that it took to report a rapid Gram stain result, another rapid test identification (PCR, FISH, etc.), a traditional identification, or a phenotypic result, the times of empirical (initial) therapy and targeted therapy, times when additional blood cultures were collected or additional laboratory or other tests were performed, and the lengths of hospital stays (days/hospital visit).
N. Other possible measures. Additional information to enhance your project results, such as total costs for the usual (comparator) and alternate (intervention) practices, antibiotic costs (targeted and/or empirical), and other information could be selected for outcome measures. Note the average cost per hospital stay (or total cost per visit), the average cost of antibiotics per visit, the percent mortality, the percentage of patients showing antibiotic resistance, the percentage of patients with hospital-acquired infections, the acute physiology and chronic health evaluation (APACHE) score (a physiological assessment based on the degree of derangement of routinely measured physiological variables that includes the APACHE score and the simplified acute physiology score [SAPS]), the sepsis-related organ failure assessment (SOFA) score (organ-specific scoring, which is similar to therapeutic scoring with the following underlying premise: the sicker a patient is, the more organ systems will be involved, ranging from organ dysfunction to failure), and any other possible measures taken (describe them).
O. Recording method. Describe how the outcomes and test results will be recorded and data collected. Describe in the final write-up any deviation from the planned method. If there is a difference in the ways the usual (comparator) practice and the alternate (intervention) practice data are recorded, indicate the recording method for each practice with the specific outcome measure(s). Additional information may be included on a separate page.
P. Statistical methods. List statistical methods to be used for the outcome measures. The basic descriptive analysis will include means, standard deviations, and confidence intervals. Additional statistical methods, such as odds ratio and risk ratio determinations, may require a statistician.

### Results/Findings

Q. Results/findings. Additional information in the QI project description of the patient population might include the diagnosis-related group (DRG) or the primary disease and comorbidities. After the quality improvement study has been completed, the next step will be to describe the findings of your study as they relate to the study design/outcome measures. For each studied outcome measure selected prior to the study, summarize the results/findings of the study/project as they relate to the practice implementation impact, i.e., the average time to the induction of targeted therapy, the LOS, the average time to report the rapid Gram stain result and/or other rapid tests, and the percent mortality. Provide the total number of observations upon which the results are based, the time period for observations, and the statistical tests performed. Discussion on whether the intervention improved outcomes relative to those of the comparator may be useful. Include calculations of the statistical significance of a difference between the measured outcomes listed in sections M and N of the usual (comparator) practice and of the alternate (intervention) practice.Basic descriptive analysis, including sample size, should be reported for each outcome. If the data are continuous (e.g., time, amount, duration), data averages (means) and standard deviations should be provided along with confidence intervals (e.g., time measures). (The means and standard deviations can be calculated in Excel using the “ = average” and “ = stdev” functions. If continuous measures are highly skewed [e.g., most values are small with a few very large values], then reporting the median and range should be reported [use Excel “ = median” and range “ = min” and “ = max”].) With dichotomous measures (e.g., mortality), there should be a numerator (number of deaths/number discharged) and denominator (number of total patients in the study) for both pre- and poststudy practices. Include findings related to cost savings if that criterion was selected for your study.Statistical assistance may be required for inferential tests of whether performance has changed significantly as a result of the alternate (intervention) practice. Additional space is available on the second page of the form (Fig. A2) for your results/findings.Examples for pre/posttest findings follow.
  - mean time to treat pretesting (comparator) = 20 min (SD = 5.5 min; *n* = 175); mean time to treat posttesting (intervention) = 12 min (SD = 3.5 min; *n* = 180);
  - median LOS pretest (comparator) = 17.3 days (range, 3 to 40 days; *n* = 1,600); median LOS posttest (intervention) = 12 days (range, 2 to 27 days; *n* = 1,250);
  - number of patients switched to targeted therapy pretest (comparator), 6 of 30 (20%); number of patients switched to targeted therapy posttest (intervention), 24 of 30 (80%);
  - mean time to targeted therapy pretest (comparator) = 10 min (SD = 4.7 min; *n* = 82); mean time to targeted therapy posttest (intervention) = 4.5 min (SD = 1.4 min; *n* = 74).
R. Barriers to implementation. Describe any external activities occurring during the quality improvement project that may have influenced the results of the project, such as staff changes or new policy. Describe organizational, personnel, technical, etc., factors that inhibited or enhanced the adoption, implementation, and/or maintenance of the practice.

## References

[1] Christenson RH, Snyder SR, Shaw CS, Derzon JH, Black RS, Mass D, Epner P, Favoretto AM, Liebow EB 2011 Laboratory medicine best practices: systematic evidence review and evaluation methods for quality improvement. Clin Chem 57:816–825. doi:10.1373/clinchem.2010.157131. 21515742

[2] Bearman GM, Wenzel RP 2005 Bacteremias: a leading cause of death. Arch Med Res 36:646–659. doi:10.1016/j.arcmed.2005.02.005. 16216646

[3] Beekmann SE, Diekema DJ, Chapin KC, Doern GV 2003 Effects of rapid detection of bloodstream infections on length of hospitalization and hospital charges. J Clin Microbiol 41:3119–3125. doi:10.1128/JCM.41.7.3119-3125.2003. 12843051 PMC165359

[4] Marchetti O, Calandra T 2002 Infections in neutropenic cancer patients. Lancet 359:723–725. doi:10.1016/S0140-6736(02)07900-X. 11888579

[5] Pittet D, Tarara D, Wenzel RP 1994 Nosocomial bloodstream infection in critically ill patients. Excess length of stay, extra costs, and attributable mortality. JAMA 271:1598–1601. 8182812 10.1001/jama.271.20.1598

[6] Klevens RM, Edwards JR, Gaynes RP 2008 The impact of antimicrobial-resistant, health care-associated infections on mortality in the United States. Clin Infect Dis 47:927–930. doi:10.1086/591698. 18752440

[7] Hall M, Levant S, DeFrances C 2013 Trends in inpatient hospital deaths: National Hospital Discharge Survey, 2000–2010. NCHS Data Brief 118. National Center for Health Statistics, Centers for Disease Control and Prevention, Atlanta, GA. 23742820

[8] Liu V, Escobar GJ, Greene JD, Soule J, Whippy A, Angus DC, Iwashyna TJ 2014 Hospital deaths in patients with sepsis from 2 independent cohorts. JAMA 312:90–92. doi:10.1001/jama.2014.5804. 24838355

[9] Elixhauser A, Friedman B, Stranges E 2006 Septicemia in U.S. hospitals, 2009: HCUP Statistical Brief 122. Agency for Healthcare Research and Quality, Rockville, MD. 22049570

[10] Beekmann SE, Diekema DJ, Doern GV 2005 Determining the clinical significance of coagulase-negative staphylococci isolated from blood cultures. Infect Control Hosp Epidemiol 26:559–566. doi:10.1086/502584. 16018432

[11] Kaye KS, Marchaim D, Chen TY, Baures T, Anderson DJ, Choi Y, Sloane R, Schmader KE 2014 Effect of nosocomial bloodstream infections on mortality, length of stay, and hospital costs in older adults. J Am Geriatr Soc 62:306–311. doi:10.1111/jgs.12634. 24438554 PMC4037885

[12] Gaieski DF, Mikkelsen ME, Band RA, Pines JM, Massone R, Furia FF, Shofer FS, Goyal M 2010 Impact of time to antibiotics on survival in patients with severe sepsis or septic shock in whom early goal-directed therapy was initiated in the emergency department. Crit Care Med 38:1045–1053. doi:10.1097/CCM.0b013e3181cc4824. 20048677

[13] Kumar A 2014 Antimicrobial delay and outcome in severe sepsis. Crit Care Med 42:e802. doi:10.1097/CCM.0000000000000620. 25402308

[14] Kumar A, Roberts D, Wood KE, Light B, Parrillo JE, Sharma S, Suppes R, Feinstein D, Zanotti S, Taiberg L, Gurka D, Kumar A, Cheang M 2006 Duration of hypotension before initiation of effective antimicrobial therapy is the critical determinant of survival in human septic shock. Crit Care Med 34:1589–1596. doi:10.1097/01.CCM.0000217961.75225.E9. 16625125

[15] Brown J, Paladino JA 2010 Impact of rapid methicillin-resistant Staphylococcus aureus polymerase chain reaction testing on mortality and cost effectiveness in hospitalized patients with bacteraemia: a decision model. Pharmacoeconomics 28:567–575. doi:10.2165/11533020-000000000-00000. 20550222

[16] Brozanski BS, Jones JG, Krohn MJ, Jordan JA 2006 Use of polymerase chain reaction as a diagnostic tool for neonatal sepsis can result in a decrease in use of antibiotics and total neonatal intensive care unit length of stay. J Perinatol 26:688–692. doi:10.1038/sj.jp.7211597. 17024143

[17] Diab M, El-Damarawy M, Shemis M 2008 Rapid identification of methicillin-resistant staphylococci bacteremia among intensive care unit patients. Medscape J Med 10:126. 18596947 PMC2438486

[18] Savinelli T, Parenteau S, Mermel LA 2004 What happens when automated blood culture instrument detect growth but there are no technologists in the microbiology laboratory? Diagn Microbiol Infect Dis 48:173–174. doi:10.1016/j.diagmicrobio.2003.10.001. 15023425

[19] Baron EJ, Weinstein MP, Dunne WM Jr, Yagupsky P, Welch DF, Wilson DM 2005 Cumitech 1C, Blood cultures IV. Coordinating ed, Baron EJ. ASM Press, Washington, DC.

[20] Peters RP, van Agtmael MA, Simoons-Smit AM, Danner SA, Vandenbroucke-Grauls CM, Savelkoul PH 2006 Rapid identification of pathogens in blood cultures with a modified fluorescence *in situ* hybridization assay. J Clin Microbiol 44:4186–4188. doi:10.1128/JCM.01085-06. 17088371 PMC1698318

[21] Arbo MD, Snydman DR 1994 Influence of blood culture results on antibiotic choice in the treatment of bacteremia. Arch Intern Med 154:2641–2645. doi:10.1001/archinte.1994.00420230024004. 7993147

[22] Bouza E, Sousa D, Munoz P, Rodriguez-Creixems M, Fron C, Lechuz JG 2004 Bloodstream infections: a trial of the impact of different methods of reporting positive blood culture results. Clin Infect Dis 39:1161–1169. doi:10.1086/424520. 15486840

[23] Schonheyder HC, Hojbjerg T 1995 The impact of the first notification of positive blood cultures on antibiotic therapy. A one-year survey. APMIS 103:37–44. 7695890 10.1111/j.1699-0463.1995.tb01077.x

[24] Weinstein MP, Murphy JR, Reller LB, Lichtenstein KA 1983 The clinical significance of positive blood cultures: a comprehensive analysis of 500 episodes of bacteremia and fungemia in adults. II. Clinical observations, with special reference to factors influencing prognosis. Rev Infect Dis 5:54–70. 6828812 10.1093/clinids/5.1.54

[25] Wellinghausen N, Kochem AJ, Disque C, Muhl H, Gebert S, Winter J, Matten J, Sakka SG 2009 Diagnosis of bacteremia in whole-blood samples by use of a commercial universal 16S rRNA gene-based PCR and sequence analysis. J Clin Microbiol 47:2759–2765. doi:10.1128/JCM.00567-09. 19571030 PMC2738079

[26] Cunney RJ, McNamara EB, Alansari N, Loo B, Smyth EG 1997 The impact of blood culture reporting and clinical liaison on the empiric treatment of bacteraemia. J Clin Pathol 50:1010–1012. doi:10.1136/jcp.50.12.1010. 9516883 PMC500382

[27] Dunagan WC, Woodward RS, Medoff G, Gray JL III, Casabar E, Smith MD, Lawrenz CA, Spitznagel E 1989 Antimicrobial misuse in patients with positive blood cultures. Am J Med 87:253–259. doi:10.1016/S0002-9343(89)80146-9. 2773963

[28] Forrest GN, Mankes K, Jabra-Rizk MA, Weekes E, Johnson JK, Lincalis DP, Venezia RA 2006 Peptide nucleic acid fluorescence in situ hybridization-based identification of Candida albicans and its impact on mortality and antifungal therapy costs. J Clin Microbiol 44:3381–3383. doi:10.1128/JCM.00751-06. 16954279 PMC1594692

[29] Fridkin SK, Edwards JR, Pichette SC, Pryor ER, McGowan JE Jr, Tenover FC, Culver DH, Gaynes RP 1999 Determinants of vancomycin use in adult intensive care units in 41 United States hospitals. Clin Infect Dis 28:1119–1125. doi:10.1086/514752. 10452645

[30] Muto CA, Jernigan JA, Ostrowsky BE, Richet HM, Jarvis WR, Boyce JM, Farr BM 2003 SHEA guideline for preventing nosocomial transmission of multidrug-resistant strains of Staphylococcus aureus and enterococcus. Infect Control Hosp Epidemiol 24:362–386. doi:10.1086/502213. 12785411

[31] Zhan C, Miller MR 2003 Excess length of stay, charges, and mortality attributable to medical injuries during hospitalization. JAMA 290:1868–1874. doi:10.1001/jama.290.14.1868. 14532315

[32] CLSI. 2007 Principles and procedures for blood cultures. Approved guidelines. CLSI, Wayne, PA.

[33] Munson EL, Diekema DJ, Beekmann SE, Chapin KC, Doern GV 2003 Detection and treatment of bloodstream infection: laboratory reporting and antimicrobial management. J Clin Microbiol 41:495–497. doi:10.1128/JCM.41.1.495-497.2003. 12517905 PMC149611

[34] Centers for Disease Control and Prevention. 2014 Rapid diagnostic tests: how they work. Centers for Disease Control and Prevention, Atlanta, GA http://www.cdc.gov/malaria/malaria_worldwide/reduction/dx_rdt.html.

[35] World Health Organization. 2014 Simple/rapid tests. World Health Organization, Geneva, Switzerland http://www.who.int/diagnostics_laboratory/faq/simple_rapid_tests/en/.

[36] Garcia LS (ed). 2010 Clinical microbiology procedures handbook, 3rd ed ASM Press, Washington, DC.

[37] Versalovic J, Carroll KC, Funke G, Jorgensen JH, Landry ML, Warnock DW (ed). 2011 Manual of clinical microbiology, 10th ed ASM Press, Washington, DC.

[38] Chorny JA, Wilson ML 1994 Rapid detection and identification of microorganisms from blood cultures. Clin Lab Med 14:181–195. 8181231

[39] Huttunen R, Syrjanen J, Vuento R, Aittoniemi J 2013 Current concepts in the diagnosis of blood stream infections. Are novel molecular methods useful in clinical practice? Int J Infect Dis 17:e934–e938. doi:10.1016/j.ijid.2013.04.018. 23871281

[40] Marlowe EM, Wolk DM 2006 Pathogen detection in the genomic era, p 505–525. *In* Tang Y (ed), Advanced techniques in diagnostic microbiology Springer Verlag, New York, NY.

[41] Pence MA, McElvania TE, Burnham CA 2013 Diagnostic assays for identification of microorganisms and antimicrobial resistance determinants directly from positive blood culture broth. Clin Lab Med 33:651–684. doi:10.1016/j.cll.2013.03.010. 23931843

[42] Riedel S, Carroll KC 2013 Laboratory detection of sepsis: biomarkers and molecular approaches. Clin Lab Med 33:413–437. doi:10.1016/j.cll.2013.03.006. 23931833

[43] Barenfanger J, Graham DR, Kolluri L, Sangwan G, Lawhorn J, Drake CA, Verhulst SJ, Peterson R, Moja LB, Ertmoed MM, Moja AB, Shevlin DW, Vautrain R, Callahan CD 2008 Decreased mortality associated with prompt Gram staining of blood cultures. Am J Clin Pathol 130:870–876. doi:10.1309/AJCPVMDQU2ZJDPBL. 19019762

[44] Institute of Medicine. 2011 Finding what works in healthcare: standards for systematic reviews. National Academies Press, Washington, DC. 24983062

[45] Borenstein M 2009 Effect sizes for continuous data, p 221–235. *In* Cooper H, Hedges L, Valentine J (ed), The handbook of research synthesis, 2nd ed Russell Sage, New York, NY.

[46] Borenstein M, Hedges L, Higgins J, Rothsteins H 2009 Introduction to meta-analysis. Chichester, West Sussex, United Kingdom.

[47] Fleiss J, Berlin J 2009 Effect size for dichotomous data, p 237–256. *In* Cooper H, Hedges L, Valentine J (ed), The handbook of research synthesis, 2nd ed Russell Sage, New York, NY.

[48] Hallin M, Maes N, Byl B, Jacobs F, De Gheldre Y, Struelens MJ 2003 Clinical impact of a PCR assay for identification of Staphylococcus aureus and determination of methicillin resistance directly from blood cultures. J Clin Microbiol 41:3942–3944. doi:10.1128/JCM.41.8.3942-3944.2003. 12904425 PMC179783

[49] Nguyen DT, Yeh E, Perry S, Luo RF, Pinsky BA, Lee BP, Sisodiya D, Baron EJ, Banaei N 2010 Real-time PCR testing for *mecA* reduces vancomycin usage and length of hospitalization for patients infected with methicillin-sensitive staphylococci. J Clin Microbiol 48:785–790. doi:10.1128/JCM.02150-09. 20071556 PMC2832423

[50] Parta M, Goebel M, Thomas J, Matloobi M, Stager C, Musher DM 2010 Impact of an assay that enables rapid determination of Staphylococcus species and their drug susceptibility on the treatment of patients with positive blood culture results. Infect Control Hosp Epidemiol 31:1043–1048. doi:10.1086/656248. 20731594

[51] Frye AM, Baker CA, Rustvold DL, Heath KA, Hunt J, Leggett JE, Oethinger M 2012 Clinical impact of a real-time PCR assay for rapid identification of staphylococcal bacteremia. J Clin Microbiol 50:127–133. doi:10.1128/JCM.06169-11. 22075595 PMC3256728

[52] Allaouchiche B, Jaumain H, Zambardi G, Chassard D, Freney J 1999 Clinical impact of rapid oxacillin susceptibility testing using a PCR assay in Staphylococcus aureus bactaeremia. J Infect 39:198–204. doi:10.1016/S0163-4453(99)90049-X. 10714795

[53] Holtzman C, Whitney D, Barlam T, Miller NS 2011 Assessment of impact of peptide nucleic acid fluorescence *in situ* hybridization for rapid identification of coagulase-negative staphylococci in the absence of antimicrobial stewardship intervention. J Clin Microbiol 49:1581–1582. doi:10.1128/JCM.02461-10. 21270213 PMC3122789

[54] Forrest GN 2007 PNA FISH: present and future impact on patient management. Expert Rev Mol Diagn 7:231–236. doi:10.1586/14737159.7.3.231. 17489730

[55] Bauer KA, West JE, Balada-Llasat JM, Pancholi P, Stevenson KB, Goff DA 2010 An antimicrobial stewardship program's impact with rapid polymerase chain reaction methicillin-resistant Staphylococcus aureus/S. aureus blood culture test in patients with S. aureus bacteremia. Clin Infect Dis 51:1074–1080. doi:10.1086/656623. 20879856

[56] Forrest GN, Roghmann MC, Toombs LS, Johnson JK, Weekes E, Lincalis DP, Venezia RA 2008 Peptide nucleic acid fluorescent in situ hybridization for hospital-acquired enterococcal bacteremia: delivering earlier effective antimicrobial therapy. Antimicrob Agents Chemother 52:3558–3563. doi:10.1128/AAC.00283-08. 18663022 PMC2565911

[57] Ly T, Gulia J, Pyrgos V, Waga M, Shoham S 2008 Impact upon clinical outcomes of translation of PNA FISH-generated laboratory data from the clinical microbiology bench to bedside in real time. Ther Clin Risk Manag 4:637–640. 18827860 10.2147/tcrm.s2838 PMC2500257

[58] Heil EL, Daniels LM, Long DM, Rodino KG, Weber DJ, Miller MB 2012 Impact of a rapid peptide nucleic acid fluorescence *in situ* hybridization assay on treatment of Candida infections. Am J Health Syst Pharm 69:1910–1914. doi:10.2146/ajhp110604. 23111676

[59] Forrest GN, Mehta S, Weekes E, Lincalis DP, Johnson JK, Venezia RA 2006 Impact of rapid in situ hybridization testing on coagulase-negative staphylococci positive blood cultures. J. Antimicrob Chemother 58:154–158. doi:10.1093/jac/dkl146. 16636084

[60] Trenholme GM, Kaplan RL, Karakusis PH, Stine T, Fuhrer J, Landau W, Levin S 1989 Clinical impact of rapid identification and susceptibility testing of bacterial blood culture isolates. J Clin Microbiol 27:1342–1345. 2473995 10.1128/jcm.27.6.1342-1345.1989 PMC267554

[61] Kerremans JJ, Verboom P, Stijnen T, Hakkaart-van Roijen L, Goessens W, Verbrugh HA, Vos MC 2008 Rapid identification and antimicrobial susceptibility testing reduce antibiotic use and accelerate pathogen-directed antibiotic use. J Antimicrob Chemother 61:428–435. 18156278 10.1093/jac/dkm497

[62] Bruins M, Oord H, Bloembergen P, Wolfhagen M, Casparie A, Degener J, Ruijs G 2005 Lack of effect of shorter turnaround time of microbiological procedures on clinical outcomes: a randomised controlled trial among hospitalised patients in the Netherlands. Eur J Clin Microbiol Infect Dis 24:305–313. doi:10.1007/s10096-005-1309-7. 15834750

[63] Doern GV, Vautour R, Gaudet M, Levy B 1994 Clinical impact of rapid *in vitro* susceptibility testing and bacterial identification. J Clin Microbiol 32:1757–1762. 7929770 10.1128/jcm.32.7.1757-1762.1994 PMC263786

[64] Drummond MF, Jefferson TO, the BMJ Economic Evaluation Working Party. 1996 Guidelines for authors and peer reviewers of economic submissions to the BMJ. BMJ 313:275–283. doi:10.1136/bmj.313.7052.275. 8704542 PMC2351717

[65] Drummond MF, O'Brien B, Stoddart G 1997 Methods for the economic evaluation of health care programmes. Oxford Medical Publications, Oxford, United Kingdom.

[66] Gabriel S, Drummond M, Maetzel A, Boers M, Coyle D, Welch V, Tugwell P 2003 OMERACT 6 Economics Working Group report: a proposal for a reference case for economic evaluation in rheumatoid arthritis. J Rheumatol 30:886–890. 12672223

[67] World Health Organization. 2011 The burden of health care-associated infection worldwide. World Health Organization, Geneva, Switzerland.

[68] O'Kane DJ, Ebert TA, Hallaway BJ, Roberts SG, Bhuiyan AK, Tenner KS 1997 A laboratorian's perspective on evaluation and implementation of new laboratory tests. Clin Chem 43:1771–1780. 9299974

[69] Rigby S, Procop GW, Haase G, Wilson D, Hall G, Kurtzman C, Oliveira K, Von Oy S, Hyldig-Nielsen JJ, Coull J, Stender H 2002 Fluorescence *in situ* hybridization with peptide nucleic acid probes for rapid identification of Candida albicans directly from blood culture bottles. J Clin Microbiol 40:2182–2186. doi:10.1128/JCM.40.6.2182-2186.2002. 12037084 PMC130801

[70] Jordan JA, Durso MB 2005 Real-time polymerase chain reaction for detecting bacterial DNA directly from blood of neonates being evaluated for sepsis. J Mol Diagn 7:575–581. doi:10.1016/S1525-1578(10)60590-9. 16258155 PMC1867550

[71] Banada PP, Chakravorty S, Shah D, Burday M, Mazzella FM, Alland D 2012 Highly sensitive detection of Staphylococcus aureus directly from patient blood. PLoS One 7:e31126. doi:10.1371/journal.pone.0031126. 22363564 PMC3281916

[72] Maaroufi Y, Heymans C, De Bruyne JM, Duchateau V, Rodriguez-Villalobos H, Aoun M, Crokaert F 2003 Rapid detection of Candida albicans in clinical blood samples by using a TaqMan-based PCR assay. J Clin Microbiol 41:3293–3298. doi:10.1128/JCM.41.7.3293-3298.2003. 12843077 PMC165319

[73] Pereira TV, Horwitz RI, Ioannidis JP 2012 Empirical evaluation of very large treatment effects of medical interventions. JAMA 308:1676–1684. doi:10.1001/jama.2012.13444. 23093165

[74] Clerc O, Prod'hom G, Senn L, Jaton K, Zanetti G, Calandra T, Greub G 2014 Matrix-assisted laser desorption ionization time-of-flight mass spectrometry and PCR-based rapid diagnosis of Staphylococcus aureus bacteraemia. Clin Microbiol Infect 20:355–360. doi:10.1111/1469-0691.12329. 23991748

[75] Clerc O, Prod'hom G, Vogne C, Bizzini A, Calandra T, Greub G 2013 Impact of matrix-assisted laser desorption ionization time-of-flight mass spectrometry on the clinical management of patients with Gram-negative bacteremia: a prospective observational study. Clin Infect Dis 56:1101–1107. doi:10.1093/cid/cis1204. 23264363

[76] Huang AM, Newton D, Kunapuli A, Gandhi TN, Washer LL, Isip J, Collins CD, Nagel JL 2013 Impact of rapid organism identification via matrix-assisted laser desorption/ionization time-of-flight combined with antimicrobial stewardship team intervention in adult patients with bacteremia and candidemia. Clin Infect Dis 57:1237–1245. doi:10.1093/cid/cit498. 23899684

[77] Martiny D, Debaugnies F, Gateff D, Gerard M, Aoun M, Martin C, Konopnicki D, Loizidou A, Georgala A, Hainaut M, Chantrenne M, Dediste A, Vandenberg O, Van Praet S 2013 Impact of rapid microbial identification directly from positive blood cultures using matrix-assisted laser desorption/ionization time-of-flight mass spectrometry on patient management. Clin Microbiol Infect 19:E568–E581. doi:10.1111/1469-0691.12282. 23890423

[78] Vlek AL, Bonten MJ, Boel CH 2012 Direct matrix-assisted laser desorption ionization time-of-flight mass spectrometry improves appropriateness of antibiotic treatment of bacteremia. PLoS One 7:e32589. doi:10.1371/journal.pone.0032589. 22438880 PMC3306318

[79] Ioannidis JP 2005 Contradicted and initially stronger effects in highly cited clinical research. JAMA 294:218–228. doi:10.1001/jama.294.2.218. 16014596

[80] Bossuyt PM, Reitsma JB, Bruns DE, Gatsonis CA, Glasziou PP, Irwig LM, Lijmer JG, Moher D, Rennie D, de Vet HC 2003 Towards complete and accurate reporting of studies of diagnostic accuracy: the STARD initiative. Standards for Reporting of Diagnostic Accuracy. Clin Chem 49:1–6. 12507953 10.1373/49.1.1

[81] Whitley E, Ball J 2002 Statistics review 4: sample size calculations. Crit Care 6:335–341. http://www.ncbi.nlm.nih.gov/pmc/articles/PMC137461/pdf/cc1521.pdf (Accessed 18 April 2014.) 12225610 10.1186/cc1521 PMC137461

